# Impact and cost-effectiveness of a lethal house lure against malaria transmission in central Côte d'Ivoire: a two-arm, cluster-randomised controlled trial

**DOI:** 10.1016/S0140-6736(21)00250-6

**Published:** 2021-02-27

**Authors:** Eleanore D Sternberg, Jackie Cook, Ludovic P Ahoua Alou, Serge Brice Assi, Alphonsine A Koffi, Dimi T Doudou, Carine J Aoura, Rosine Z Wolie, Welbeck A Oumbouke, Eve Worrall, Immo Kleinschmidt, Raphael N'Guessan, Matthew B Thomas

**Affiliations:** a Department of Entomology, Center for Infectious Disease Dynamics, The Pennsylvania State University, University Park, PA, USA; b Department of Vector Biology, Liverpool School of Tropical Medicine, Liverpool, United Kingdom; c Innovative Vector Control Consortium, Liverpool School of Tropical Medicine, Liverpool, United Kingdom; d Department of Infectious Disease Epidemiology, London School of Hygiene & Tropical Medicine, London, UK; e Department of Disease Control, London School of Hygiene & Tropical Medicine, London, UK; f Institut Pierre Richet, Institut National de Santé Publique, Bouaké, Côte d'Ivoire; g Laboratoire de Santé, Nutrition et Hygiène, Centre de Recherche pour le Développement, Université Alassane Ouattara, Bouaké, Côte d'Ivoire; h Laboratoire de genetique, Unité de Formation et de Recherche en Biosciences, Université Felix Houphouët Boigny, Abidjan, Côte d'Ivoire; i Wits Research Institute for Malaria, School of Pathology, University of the Witwatersrand, Johannesburg, South Africa; j Southern African Development Community Malaria Elimination Eight Secretariat, Windhoek, Namibia; k York Environmental Sustainability Institute and Department of Biology, University of York, York, UK

## Abstract

**Background:**

New vector control tools are required to sustain the fight against malaria. Lethal house lures, which target mosquitoes as they attempt to enter houses to blood feed, are one approach. Here we evaluated lethal house lures consisting of In2Care (Wageningen, Netherlands) Eave Tubes, which provide point-source insecticide treatments against host-seeking mosquitoes, in combination with house screening, which aims to reduce mosquito entry.

**Methods:**

We did a two-arm, cluster-randomised controlled trial with 40 village-level clusters in central Côte d'Ivoire between Sept 26, 2016, and April 10, 2019. All households received new insecticide-treated nets at universal coverage (one bednet per two people). Suitable households within the clusters assigned to the treatment group were offered screening plus Eave Tubes, with Eave Tubes treated using a 10% wettable powder formulation of the pyrethroid β-cyfluthrin. Because of the nature of the intervention, treatment could not be masked for households and field teams, but all analyses were blinded. The primary endpoint was clinical malaria incidence recorded by active case detection over 2 years in cohorts of children aged 6 months to 10 years. This trial is registered with ISRCTN, ISRCTN18145556.

**Findings:**

3022 houses received screening plus Eave Tubes, with an average coverage of 70% across the intervention clusters. 1300 eligible children were recruited for active case detection in the control group and 1260 in the intervention group. During the 2-year follow-up period, malaria case incidence was 2·29 per child-year (95% CI 1·97–2·61) in the control group and 1·43 per child-year (1·21–1·65) in the intervention group (hazard ratio 0·62, 95% CI 0·51–0·76; p<0·0001). Cost-effectiveness simulations suggested that screening plus Eave Tubes has a 74·0% chance of representing a cost-effective intervention, compared with existing healthcare activities in Côte d'Ivoire, and is similarly cost-effective to other core vector control interventions across sub-Saharan Africa. No serious adverse events associated with the intervention were reported during follow-up.

**Interpretation:**

Screening plus Eave Tubes can provide protection against malaria in addition to the effects of insecticide-treated nets, offering potential for a new, cost-effective strategy to supplement existing vector control tools. Additional trials are needed to confirm these initial results and further optimise Eave Tubes and the lethal house lure concept to facilitate adoption.

**Funding:**

The Bill & Melinda Gates Foundation.

## Introduction

Widescale implementation of core vector control—specifically long-lasting insecticidal nets and indoor residual spraying—has contributed to substantial reductions in the burden of malaria.^1^, ^2^ However, these reductions have plateaued^2^ (probably due to numerous causes, including insecticide resistance, poor availability or misuse of bednets, urbanisation, and limits on donor funding), and new control strategies are needed to attain the milestones laid out in the WHO Global Technical Strategy for malaria control.^3^, ^4^, ^5^

**Figure 1:**
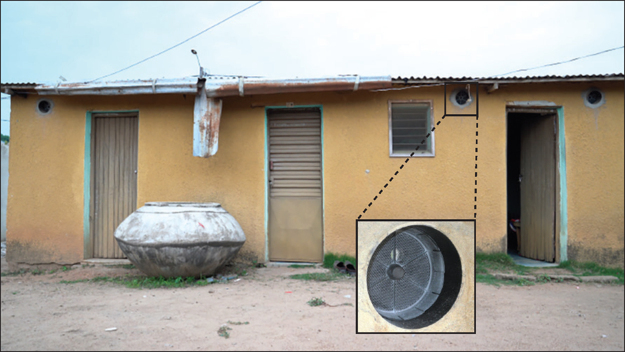
A house in Côte d'Ivoire with screening plus Eave Tubes The inset shows the Eave Tube insert placed within the tube.

One growing area of interest is the potential role of housing improvements.^6^, ^7^ Many traditional house designs in Africa have open eaves (the area where the wall joins the roof of a house). Studies have shown that closing the eaves can reduce indoor vector abundance^8^, ^9^ and that closed eaves, together with modern housing elements, such as metal roofs, window screening, and improved doors, are associated with reduced malaria burdens.^10^, ^11^, ^12^, ^13^ There is also a body of work exploring the potential to supplement physical barriers by adding insecticides.^14^, ^15^, ^16^ The In2Care (Wageningen, Netherlands) Eave Tube is one such approach, consisting of ventilation tubes made from pieces of polyvinyl chloride (PVC) pipe embedded in a closed eave.^17^ Each tube holds a removable insert with electrostatically charged netting that can hold powder formulations of insecticides (figure 1). Mosquitoes are drawn into the Eave Tubes, which funnel the heat and odour cues that normally emanate from the eaves of an occupied house. Laboratory bioassays have shown that Eave Tube inserts can transfer a high dose of insecticide powder to mosquitoes and are even lethal to insecticide-resistant mosquitoes.^18^ Semi-field studies in Tanzania,^19^ Kenya,^20^ and Côte d'Ivoire^21^, ^22^ have shown attraction of mosquitoes to the tubes and reduced overnight survival.

**Research in context**

**Evidence before this study**

We searched PubMed on May 4, 2020, using the search term “malaria” in combination with “housing”, “lethal house lure”, or “eaves”, with no restriction on language or dates. The relevant results from this search were primarily observational studies aimed at identifying risk factors associated with malaria. There were also systematic reviews and meta-analyses of survey data collected in multiple African countries during Demographic and Health Surveys and Malaria Indicator Surveys. These studies typically found a reduced risk of malaria for people living in improved housing (ie, housing with metal roofs, cement floors, and closed eaves). However, few studies tested house improvement as an intervention for malaria control, and none at the village scale. Of 675 search results, there were only four randomised controlled trials that evaluated house modifications, all of which were at household level, and only three included health outcomes (clinical malaria, parasitaemia, or anaemia). These trials evaluated insecticide-treated curtains (in Kenya in 1990–91) or house screening (in The Gambia in 2006–07 and in Ethiopia in 2015–16). In all three trials, there was a positive effect on entomological and health outcomes in houses that received the intervention compared with control houses. For example, the trial in The Gambia found that mean mosquito density was 59% lower in households with full screening than in households without screening and 47% lower in households with screened ceilings. The odds of anaemia in children was 47% lower in households with full screening and 49% lower in households with screened ceilings. However, the trial reported no difference in parasitaemia.

**Added value of this study**

This study is the first cluster-randomised controlled trial to evaluate screening plus In2Care (Wageningen, Netherlands) Eave Tubes, a new intervention classed as a lethal house lure by WHO. To the best of our knowledge, it is the only cluster-randomised controlled trial that has evaluated combining house improvements with targeted insecticide treatment, and it is the only village-level trial evaluating house interventions. It is also one of a small number of any type of trial evaluating house modifications for malaria control.

**Implications of all the available evidence**

Even without gold standard evidence from randomised controlled trials, the idea of mosquito-proof housing for malaria control can be traced back more than a century. However, the first systematic review of housing and malaria was published just 5 years ago, and a Cochrane Review of housing and malaria was completed in January, 2021 (protocol published in 2020). This cluster-randomised controlled trial adds to that evidence base, as the first evaluation of screening plus Eave Tubes as a lethal house lure approach and Eave Tubes as a novel vector control tool. With issues such as insecticide resistance, low coverage and usage, and unreliable durability compromising the efficacy of long-lasting insecticidal nets and indoor residual spraying, new tools and interventions like Eave Tubes and screening plus Eave Tubes are essential to meet malaria elimination milestones. The results of this trial justify screening plus Eave Tubes as a cost-effective malaria control intervention that works in an area where malaria vectors are highly resistant to insecticide.

We combined Eave Tubes with mosquito-proofing of houses (ie, screening of windows and closing gaps) to create what WHO describes as a lethal house lure^23^ for host-seeking mosquitoes. We refer to this new intervention as screening plus Eave Tubes (SET). We expected the screening component to provide a physical barrier to reduce household entry and the Eave Tubes to increase mosquito mortality; thus, SET could provide community level protection at sufficiently high coverage.^24^ We aimed to evaluate the epidemiological and entomological effects of SET, and its cost-effectiveness, in the presence of universal coverage of insecticide-treated nets.

## Methods

### Study design and participants

We did a two-arm, cluster-randomised controlled trial with 20 clusters per group in the Gbêkê region in central Côte d'Ivoire. This region has year-round malaria transmission, with a peak during the wet season (May–October). The local malaria vector populations are highly resistant to almost all classes of insecticides used for vector control.^25^ The *Anopheles gambiae* species complex is the dominant species, with *A funestus* and *A nili* also present.^26^ We have previously published a detailed description of the study protocol.^27^

We defined clusters as villages separated by at least 2 km, with 100–600 houses and at least 80% of the houses appearing suitable for modification with SET (ie, roofs made from metal sheeting and walls made from concrete or brick) during our initial visit.

Between July 10 and July 30, 2016, we did a census in all clusters to collect details of household members (age and sex), number of insecticide-treated nets available to households (provided during a national distribution campaign in 2014), and structure of houses. From the census list, we randomly selected 60 children (aged 6 months to 10 years) from each cluster for a baseline survey between Aug 4 and Aug 26, 2016. The survey collected data on malaria infection status, net use, and household assets as a proxy for socioeconomic status. All the households in the clusters that were assigned to SET (intervention clusters) were offered SET if the structure of their house was suitable. All households in the intervention and control clusters were offered insecticide-treated nets, regardless of their participation in any other aspect of the trial.

We measured epidemiological impact through active case detection, with clinical teams systematically screening for malaria, in a cohort of 50 randomly selected children (aged 6 months to 10 years for the duration of the follow-up) per cluster. We measured entomological effects using human landing catches every month for 2 years in randomly selected households.

The trial was reviewed and approved by the Côte d'Ivoire Ministry of Health ethics committee (039/MSLS/CNER-dkn), the Pennsylvania State University's Human Research Protection Program under the Office for Research Protections (STUDY00003899 and STUDY00004815), and the London School of Hygiene & Tropical Medicine ethical review board (11223). We obtained written and verbal informed consent from all trial participants or guardians for participants younger than 18 years. A trial steering committee monitored trial progress and adherence to protocol.

### Randomisation and masking

We used restricted randomisation to ensure balanced cluster allocation with respect to cluster size, proportion of households suitable for the intervention, and presence of a health facility within a cluster, as well as to household socioeconomic status, malaria infection prevalence in cohort aged children, and insecticide-treated net use. We generated 10 000 potential sequences for the randomisation using Stata (version 15), one of which was picked by random drawing during a public ceremony on Sept 7, 2016.

Given the visible nature of SET, it was impossible to conceal the cluster allocation from the participants or the field workers. However, cluster allocation was masked for the processing of samples and all data analyses were blinded.

### Procedures

In the houses that received SET, work teams installed locally sourced PVC tubes (20 cm long with a diameter of 15 cm) around 20 cm below the roof at 1·5–2·0 m intervals across the outside walls of occupied rooms, such as bedrooms and living rooms, but not storage rooms. The teams used brick, cement, and plaster to seal open eaves and other obvious gaps in the walls and screened the windows using custom-built wooden frames with untreated UV-resistant PVC-plasticised glass-fibre enforced netting, consisting of 18 × 14 density yarns of 130 g/m^2^ (Copaco Screenweavers; Harelbeke, Belgium).

During the installations, work teams placed untreated Eave Tube inserts (In2Care; Wageningen, Netherlands) into the PVC tubes. After the installations were completed in all SET clusters, work teams replaced the untreated inserts with inserts treated using a 10% wettable powder formulation of β-cyfluthrin (Tempo Ultra WP; Bayer; Cary, NC, USA). β-cyfluthrin is a pyrethroid with existing regulatory approval in Côte d'Ivoire and was selected for this trial on the basis of initial assays that showed 100% mortality against local wild-type mosquitoes using Eave Tubes.^22^

SET was monitored by teams walking through villages every 4 months, and repairs were made as required. Every month, the teams collected a random sample of four inserts in each SET cluster to monitor persistence of the insecticide. Mosquito mortality was assessed using Eave Tube bioassays^22^ with field-collected mosquitoes. When inserts from any village produced mortality of 80% or less, teams began replacing inserts in all the SET clusters with freshly treated inserts to ensure that the replacement process could be completed before reaching the 70% mortality threshold specified in the protocol.

We completed an insecticide-treated net distribution campaign (polyester nets treated with 1·8 g/kg deltamethrin; PermaNet 2.0; Vestergaard; Lausanne, Switzerland) in all study clusters between March 8 and March 19, 2017, according to Côte d'Ivoire's National Malaria Control Program guidelines.

**Figure 2:**
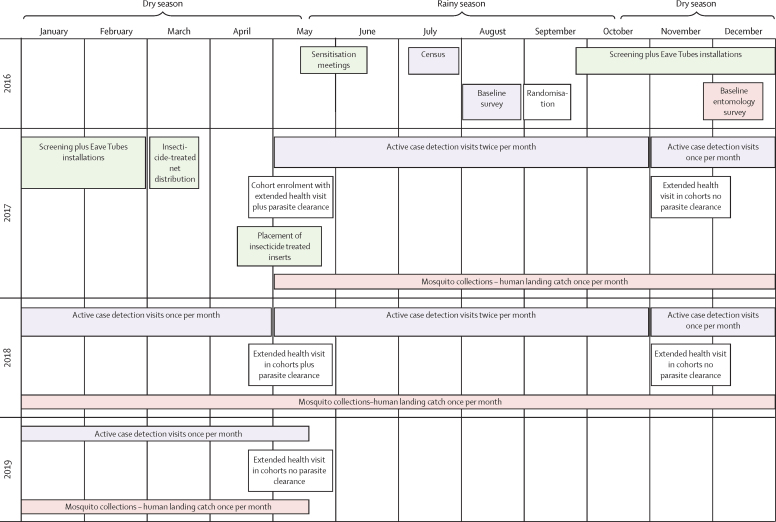
Timeline of study activities

Once the installation of SET and distribution of insecticide-treated nets were completed we began epidemiological and entomological monitoring (figure 2). The active case detection cohort children were monitored for 2 years unless parents withdrew consent, or the child moved out of the cluster. Children lost to follow-up were replaced with another child of similar age from the same cluster, preferably from the same household or a neighbouring household, using the same enrolment procedure. Study nurses visited children in the active case detection cohort, under the supervision of a medical doctor and with the assistance of community health workers. At the initial enrolment visit, all children received a 3-day course of a first-line antimalarial (artesunate-amodiaquine or artemether-lumefantrine), to clear any existing malaria parasite infection. A second round of parasite clearance took place 1 year later.

At each active case detection visit—once per month during the dry season (November–April) and twice per month during the rainy season (May–October)—the clinical team recorded the axillary temperature of each child. If the child was febrile (axillary temperature ≥37·5°C) or had a history of fever in the past 48 h or the parents reported that their child was sick, the child received a physical examination and a record was made of symptoms, pulse, and respiratory rate. A finger prick blood sample was taken from all febrile children for a malaria rapid diagnostic test (SD Bioline Malaria Ag P.f/Pan; Standard Diagnostics; Seoul, South Korea). If the rapid diagnostic test was positive, the child was treated with an antimalarial according to national guidelines.

At the start and end of the rainy season (April and November) in both study years, all children in the cohort received a general physical exam to check for any other symptoms, particularly respiratory symptoms. We also measured haemoglobin concentrations in children aged 5 years and younger using a spectrophotometer (HemoCue Hb 201+; Radiometer Medical; Ängelholm, Sweden) to measure the prevalence of anaemia.

For the human landing catch sampling, volunteers were recruited from the study clusters to capture mosquitoes. Volunteers sat with their legs uncovered from 18:00 to 08:00, trapping any mosquitoes that landed on their legs in glass haemolysis tubes plugged with cotton. Capturers worked in two shifts, from 18:00 to 01:00 and from 01:00 to 08:00, and were supervised by research technicians to ensure they were working according to protocol. Catches were done indoors and outdoors for one night per month in four randomly selected houses per cluster.

Technicians brought the mosquitoes back to the laboratory for morphological species identification. A random subset of malaria vectors was dissected to assess parity. We used PCR to assess sporozoite prevalence in a random sample of up to 60 parous females per cluster per sampling night (non-parous females were classed as uninfected since they had not yet taken a bloodmeal).

We extracted economics data from project expenditure records and ethnographic studies of six villages in the trial. Costs were recorded in the currency of expenditure (euros or West African francs). West African francs were converted to euros and then to US dollars using mean exchange rates for the period Sept 1, 2016, to April 02, 2019 (appendix p 5). Capital items (replacement value for one unit >€500 euros and a useful life >1 year) were annualised over useful life in the financial analysis and at a discount rate of 3% in the economic analysis. The value of donated resources (insecticide and community labour) was imputed from market rates. We attributed costs to initial screening and housing modification, Eave Tube installation, insecticide treatment and retreatment rounds (comprising insert treatment, insert cleaning, and insert insertion and replacement), and housing maintenance rounds.

At the end of the trial, we did a cost-effectiveness analysis to measure the incremental economic and financial cost per malaria case averted and the cost per disability-adjusted life year (DALY) averted with SET plus insecticide-treated nets compared with insecticide-treated nets alone, under trial conditions from the societal (provider and community) and provider perspectives. Trial costs and the between-group difference in malaria case incidence were combined with malaria case fatality rate estimates and population data from the trial and other sources to simulate cost-effectiveness endpoints, with DALY averted calculated according to standard methods.^28^ We used Monte Carlo simulation in @Risk Software version 7.5 to explore uncertainty. Further details on the economic methods are provided in the appendix (pp 4–7).

### Outcomes

The primary outcome of the trial was the incidence of clinical malaria (axillary temperature ≥37·5°C and positive rapid diagnostic test) in children enrolled in the active case detection cohort. The secondary clinical outcome was the prevalence of anaemia in children aged 5 years and younger (moderate anaemia was defined as 7·0–9·9 g/dL haemoglobin and severe anaemia as <7·0 g/dL haemoglobin). Adverse events were also recorded, including skin irritations, headaches, fatigue, diarrhoea, nausea, and moderate to severe respiratory symptoms. Any deaths in the cohort were followed up with a verbal autopsy by a medical doctor if the parents consented.

The key entomological outcomes were the number of malaria vectors captured per person per night both indoors and outdoors by human landing catches and the entomological inoculation rate (EIR; number of infective bites per person per year). The key economic outcomes were the incremental economic and financial cost per malaria case averted, and cost per DALY averted with SET plus insecticide-treated nets compared with insecticide-treated nets alone.

Other planned secondary outcomes (malaria infection in children in the cohorts, parity in malaria vectors, and user behaviour, perceptions, and acceptability relating to SET) and a supplementary cross-sectional survey that measured prevalence of malaria in the study area will be reported in future publications.

### Statistical analysis

The sample size calculations for epidemiological and entomological data collection are published.^27^ Briefly, we designed the study to detect a 40% difference in malaria case incidence, assuming a control group incidence of 0·5 malaria cases per child-year and a coefficient of variation of 0·5 between clusters. This design required 20 clusters per group and 50 children per cluster followed for 2 years.

For the entomological sampling, we powered the study to detect a 50% reduction in mosquito density, consistent with semi-field testing of Eave Tubes^19^, ^20^ and other randomised controlled trials evaluating the entomological effect of house modifications.^12^, ^29^ On the basis of baseline data, this sample size calculation required seven houses per cluster for the human landing catch captures. To capture seasonal variation, we repeated sampling throughout the trial. For logistical reasons, we did human landing catch captures in four randomly selected houses every month and pooled the data every 2 months.

We used Stata (version 15) to analyse epidemiological and entomological data. The primary intention-to-treat analysis was a comparison of the incidence of clinical malaria episodes between the two groups. We considered that a child would not be at risk for 2 weeks following any treatment. Malaria cases detected within 4 weeks of a previous malaria case were not counted as a new case, to allow for circulating histidine-rich protein after parasite clearance.^30^ Per protocol analyses involved comparison of children who lived in houses with SET installed with those in the control clusters. Sensitivity analysis included increasing the censoring period following treatment to 4 weeks or requiring at least one negative visit following a positive diagnosis before follow-up time was uncensored. Children were allowed to drop out and re-enter the cohort if they were away from their home village for an extended period of time (eg, for school holidays); if a child was not visited for a period of more than 6 weeks, we did not include this period in the follow-up time. We attempted to replace children lost from the cohort with children of a similar age from the same household, if possible, or from a randomly selected neighbouring household. We also assessed the effect of year and possible confounders such as age, socioeconomic status, and cluster baseline infection prevalence.

We used survival analysis to compare the risk of having a malaria case in each group using a Cox proportional hazards model allowing for multiple events per child and using robust estimates of variance to account for the clustered design. The effect of the intervention on anaemia was estimated using mixed effects logistic models with child included as a random effect to account for repeated measures and robust standard errors.

We calculated a proxy for socioeconomic status for each child, incorporating information about household wall type, toilet type, access to electricity, access to water, and household assets (bicycle, motorbike, television, radio, refrigerator, and livestock) into a principal components analysis. The subsequent socioeconomic status score was divided into tertiles. To assess the effect of SET coverage, we compared villages with SET coverage of more than 70% coverage and of 70% or less, with control villages using Cox regression. We used the 70% threshold on the basis of modelling outputs showing community-wide benefits at this threshold^24^ and to allow a balanced number of clusters on either side of the threshold. We used the same Cox regression model described above to model the relationship between SET coverage and incidence in the intervention clusters, whilst controlling for baseline cluster prevalence.

Indoor and outdoor mosquito density (malaria vectors only) was calculated for each household visit. A pooled EIR was calculated for each cluster at each visit (12 visits total). All non-parous mosquitoes were assumed to be sporozoite negative. EIR was calculated as follows:

$$\begin{array}{c} AnnualEIR=\frac{Totalvectorscaughtbyhumanlandingcatch}{Totalcapturenights}\\ \times \frac{Totalsporozoitepositive}{Totaltested+Totalnonparous}\times 365 \end{array}$$

To analyse mosquito density and EIR, we used mixed effects generalised linear mixed models with a negative binomial distribution, controlling for collection timepoint as a categorical fixed effect. Cluster was included in the model as a random effect.

This study is registered as an International Standard Randomised Controlled Trial, ISRCTN18145556.

### Role of the funding source

The funder of the study had no role in study design, data collection, data analysis, data interpretation, or writing of the report. EDS and JC had full access to all the data in the study, and EDS had final responsibility for the decision to submit for publication. In2Care (the technology provider) was involved in putting together the grant proposal to secure funding from the Bill & Melinda Gates Foundation for the trial. In2Care also installed and maintained the technology (screening plus Eave Tubes) throughout the trial. The study design, conduct, analysis, and write up was done independently of In2Care, by the authors named on the paper.

## Results

**Figure 3:**
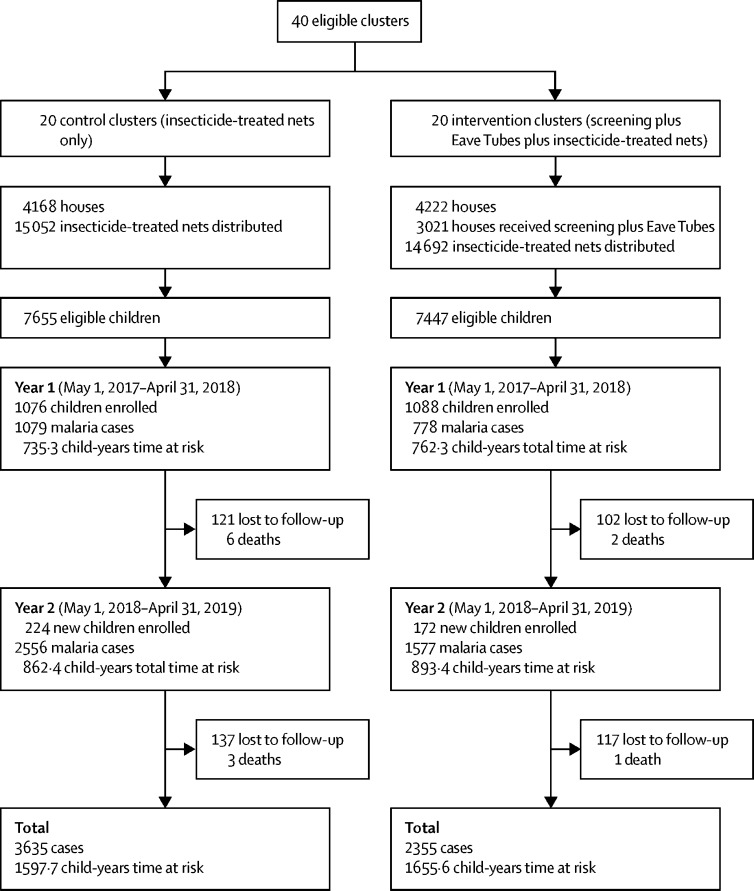
Study profile

The study area included 8390 houses with 55 404 inhabitants at the time of the census (figure 3). We installed SET from Sept 26, 2016, to Feb 26, 2017, and installed treated inserts between April 15 and May 22, 2017, before enrolment of children in the village. There were 4222 houses in the intervention group, of which 3021 (72%) received SET, resulting in cluster-level coverage ranging from 32% to 100% (median 73%). 15 052 insecticide-treated nets were distributed in the control group and 14 692 in the intervention group, with an average of one net for every 1·9 people in the control group and every 1·8 people in the intervention group (figure 3). The groups did not differ on cluster size, previous net ownership, children living in houses suitable for SET, or malaria infection prevalence (table 1).

**Table 1:** Baseline characteristics

		**Control group**	**Intervention group**
**Cluster characteristics (July, 2016)**			
Number of clusters		20	20
Mean number of houses per cluster (range)		201 (106–354)	205 (102–353)
Mean population per cluster (range)		1365 (598–2382)	1351 (655–2332)
Percentage of households with adequate numbers of long-lasting insecticidal nets (95% CI)*		39·5% (34·1–44·8)	43·6% (36·6–50·6)
**Baseline cross-sectional (August, 2016)**			
Mean number of children aged 6 months to 10 years surveyed per cluster (range)		63 (52–94)	64 (50–95)
Mean age, years (range)		4·6 (0·5–9·6)	4·7 (0–8·8)
Percentage of children living in houses of suitable structure for screening plus Eave Tubes (95% CI)		87·9% (80·7–95·1)	92·3% (88·2–96·4)
Malaria infection prevalence (95% CI)		73·9% (68·1–79·7)	72·4% (66·4–78·3)
**Entomological characteristics—human landing catches (December, 2016)**			
Mean number of vectors found indoors per house per night (95% CI), n		29·8 (14·3–45·3), 3575	25·8 (6·4–45·2), 2836
Mean number of vectors found outdoors per house per night (95% CI), n		26·4 (13·7–39·2), 3173	21·6 (4·4–38·9), 2381
**Characteristics of children at enrolment (April–May, 2017)**			
Age, percentage (95% CI)			
	≤2 years	9·4% (7·4–11·8)	9·7% (7·6–12·3)
	>2–5 years	38·4% (34·6–42·3)	35·6% (32·3–39·1)
	>5–8 years	52·3% (48·0–56·5)	54·7% (51·2–58·2)
Sex, percentage (95% CI)			
	Male	50·5% (47·5–53·5)	50·1% (47·8–52·3)
	Female	49·5% (46·5–52·6)	49·7% (47.7–52.2)
Proportion of children reporting using a net the night before enrolment (95% CI)		58·0% (43·9–72·1)	55·8% (42·4–69·1)
Proportion of children living in houses with screening plus Eave Tubes (95% CI)		..	82·5% (77·1–87·9)
Malaria case prevalence (95% CI)		3·2% (1·2–5·3)	4·2% (1·7–6·6)
Anaemia prevalence in children ≤5 years† (95% CI)		40·6% (28·5–52·7)	36·5% (28·8–44·2)

CIs are calculated at the individual (epidemiological) or household (vector density) level and adjusted for clustering by village.

* One net for every two people.

† Anaemia was clinically diagnosed as <9·9 g/dL.

We recruited 1300 children in the control group and 1260 in the intervention group over the 2-year period for active case detection, giving a follow-up time of 3253 child-years. At enrolment, children were balanced on age, sex, and net use. A similar proportion of children were febrile and had detectable malaria parasites at enrolment in each group (33 [3·2%] of 1022 in the control group *vs* 43 [4·2%] of 1031 in the intervention group). Entomological data collected between Nov 24 and Dec 17, 2016 (before installation of treated inserts), showed fewer vectors in the intervention group both indoors and outdoors (table 1).

**Figure 4:**
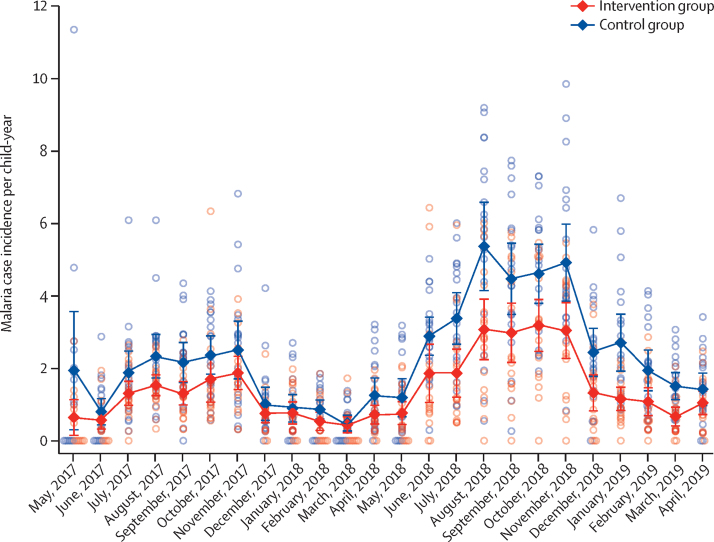
Cluster-level malaria case incidence by month for the 2-year follow-up Open circles show cluster-level malaria case incidence, and closed diamonds show the mean of the cluster incidences, with bars representing 95% CIs.

During the 2-year follow-up period, bednet use the previous night was reported at 24 270 (67·2%) of 36 095 visits for the control group and at 21 428 (59·0%) of 36 316 visits for the intervention group. We detected 5990 malaria cases. Malaria case incidence was 2·29 per child-year (95% CI 1·97–2·61) in the control group and 1·43 per child-year (1·21–1·65) in the intervention group (hazard ratio [HR] 0·62, 95% CI 0·51–0·76; p <0·0001; table 2). Malaria case incidence was higher in both groups in the second study year, with a slightly larger difference between the groups (0·60, 0·47–0·78; p <0·0001) compared with year 1 (0·70, 0·59–0·82; p<0·0001; figure 4; table 2). Interactions between covariates (age, study year, socioeconomic status, or baseline prevalence) and group were not significant.

**Table 2:** Malaria case incidence in children aged 6 months to 10 years

	**Control group**			**Intervention group**			**Hazard ratio**	**p value**
	Number of clinical malaria episodes	Follow-up time, child-years	Incidence per child-year (95% CI)	Number of clinical malaria episodes	Follow-up time, child-years	Incidence per child-year (95% CI)		
Overall	3635	1597·7	2·29 (1·97–2·61)	2355	1655·6	1·43 (1·21–1·65)	0·62 (0·51–0·76)	<0·0001
Year 1	1079	735·3	1·47 (1·28–1·67)	778	762·3	1·02 (0·92–1·12)	0·70 (0·59–0·82)	<0·0001
Year 2	2556	862·4	3·00 (2·52–3·48)	1577	893·4	1·79 (1·41–2·17)	0·60 (0·47–0·78)	<0·0001

Hazard ratios derived from Cox regression, with robust standard errors for cluster, are presented.

Overall prevalence of anaemia reduced from 38·5% (346 of 898) at baseline to 14·5% (108 of 747) 6 months later and remained similar throughout follow-up. The odds of anaemia in children living in intervention clusters was 30% lower than those living in control clusters (odds ratio 0·69, 95% CI 0·49–0·99; p=0·046; table 3). Only ten instances of severe anaemia were recorded during the study period.

**Table 3:** Prevalence of anaemia in children aged 6 months to 10 years

	**Control group**		**Intervention group**		**Odds ratio**	**p value**
	Number of anaemia tests	Prevalence of anaemia (95% CI)	Number of anaemia tests	Prevalence of anaemia (95% CI)		
Overall	1171	16·2% (13·9–18·6)	1162	12·1% (9·2–15·0)	0·69 (0·49–0·99)	0·046
Year 1	621	17·4% (13·8–21·0)	643	13·4% (9·4–17·4)	0·70 (0·43–1·15)	0·16
Year 2	550	14·9% (12·0–17·9)	519	10·6% (7·3–13·8)	0·59 (0·34–1·02)	0·061

Haemoglobin tests for anaemia were done at the start and end of each transmission season in children aged 5 and younger. The first study year includes anaemia tests done 7 months after intervention. The second year includes tests done at 12 months, 19 months, and 24 months after intervention. Odds ratios derived from mixed effect logistic regression, with a random effect for child and robust standard errors for cluster, are presented for prevalence of anaemia.

**Figure 5:**
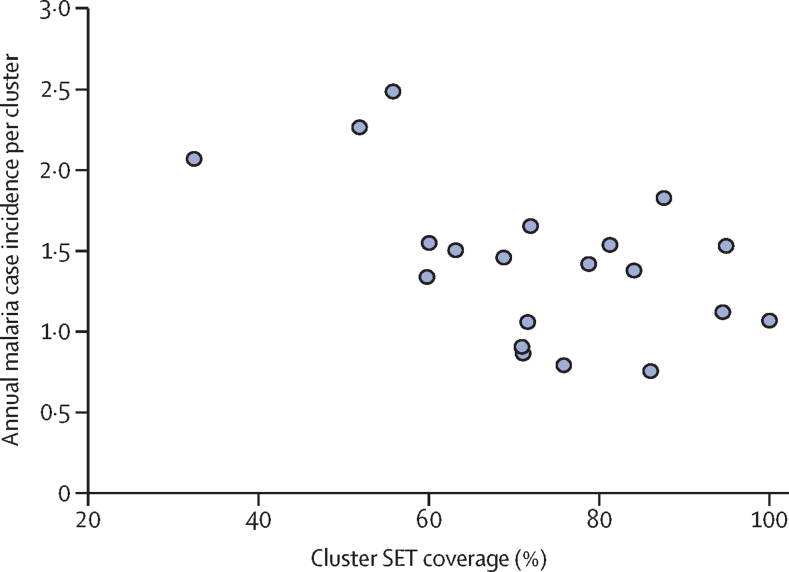
Association between SET coverage and malaria case incidence Each circle represents a cluster. SET=screening plus Eave Tubes.

The range in SET coverage enabled an exploration of the potential dose-response effect of intervention coverage. However, such an analysis was not included in the analysis plan, and the trial was not powered for this purpose, so insights into coverage should be treated with caution. In clusters where coverage was greater than 70% (13 clusters), risk of a malaria case was 47% lower compared with control clusters (HR 0·53, 95% CI 0·43–0·65; p<0·0001). In clusters with 70% coverage or less (seven clusters), there was some benefit compared with control villages (0·79, 0·63–1·00; p=0·050). There was a strong association between incidence and SET coverage, with a 10% decrease in incidence for every 10% increase in SET coverage, controlling for baseline prevalence (0·90, 0·52–0·94; p<0·0001; figure 5).

Per-protocol analysis (ie, only including children living in households that received nets in the control group and nets and SET in the intervention group) showed a slightly higher effect than the intention-to-treat analysis (HR 0·59, 95% CI 0·48–0·72; p<0·0001). 214 (17%) of 2160 children in the intervention group were living in houses without SET, which enabled us to examine whether those children benefitted from a community effect. Because children that did not live in houses with SET were more likely to live in houses not suitable for the intervention (often associated with lower socioeconomic status), we included socioeconomic status tertile as a fixed effect in the model. There was evidence that children living in intervention clusters, but without the intervention, were at lower risk of having a case of malaria than those living in control clusters (HR 0·73, 95% CI 0·54–0·99; p=0·042). This effect was not present in clusters where SET coverage was 70% or less (0·96, 0·78–1·19; p=0·73).

Respiratory infections were rare, with no difference between the two groups (HR 1·00, 95% CI 0·71–1·41; p=0·99). Other adverse events recorded are noted in the appendix (p 2). No serious adverse events associated with the intervention were reported during the trial. During the 2-year follow-up, 12 children died (nine in the control group, and three in the intervention group). Five of the deaths were caused by malaria (three in the control group, and two in the intervention group).

Mean indoor mosquito density was substantially reduced in the intervention group (24·7 [95% CI 15·1–34·3] in the control group *vs* 13·0 [5·9–20·1] in the intervention group) with mosquito density ratio of 0·39 (95% CI 0·20–0·74; p=0·0040). There was also some evidence of a reduction in mean outdoor mosquito density (mosquito density ratio 0·61, 95% CI 0·34–1·10; p=0·099; table 4).

**Table 4:** Entomological outcomes by intervention and location for malaria vectors

	**Control group**	**Intervention group**	Mosquito density ratio*(95% CI)	Rate ratio*(95% CI)	**p value**
**Indoors**					
Mean mosquito density (95% CI), total collected	24·7 (15·1–34·3), 60 393	13·0 (5·9–20·1), 31 724	0·39 (0·20–0·74)	..	0·0040
Entomological inoculation rate (95% CI), total tested for sporozoites	170 (131–210), 7911	53 (34–71),5274	..	0·28 (0·15–0·50)	<0·0001
**Outdoors**					
Mean mosquito density (95% CI), total collected	28·0 (18·5–37·4), 68 237	20·6 (10·4–30·8), 50 311	0·61 (0·34–1·10)	..	0·099
Entomological inoculation rate (95% CI), tested for sporozoites	165 (123–206), 8261	61 (38–96), 6518	..	0·33 (0·19–0·57)	<0·0001

* Adjusted for collection visit.

EIR was substantially reduced in the intervention group both indoors and outdoors. Indoor annual EIR was 170 infectious bites per person (95% CI 131–210) in the control group and 53 infectious bites per person (34–71) in the intervention group (rate ratio [RR] 0·28, 95% CI 0·16–0·50; p<0·0001). Outdoor EIR was 165 infectious bites per person (95% CI 123–206) in the control group and 61 infectious bites per person (38–96) in the treatment group (RR 0·33, 95% CI 0·19–0·57; p<0·0001).

Total economic costs of the intervention were US$239·46 per house covered from the societal perspective and $215·38 per house from the provider perspective (appendix pp 9, 25). The total annualised societal-perspective economic costs of delivering screening and Eaves Tube was $723 421·06, with 96·7% of these costs ($699 388·64 of $723 421·06) attributed to non-capital items and 3·3% ($24 032·42 of $723 421·06) attributed to capital items (appendix pp 8, 19). Providers bore 89·9% of these costs ($650 676·72 of $723 421·06), with the remaining 10·1% borne by communities (appendix pp 8, 19). Total costs were comprised of 40·0% for screening installation ($289 566·13 of $723 421·06), 39·5% for Eave Tubes installation ($285 537·93 of $723 421·06) and 20·5% for the six maintenance and retreatment rounds ($148 317·01 of $723 421·06; appendix pp 8, 21).

Cost-effectiveness simulations using model inputs (appendix pp 17–18) indicated the estimated median societal cost per case averted was $28·91 per year (90% credible interval 6·82–74·21) and median cost per DALY averted was $210·29 per year (46·16–553·57). The equivalent provider-perspective cost-effectiveness estimates for cost per case averted was $26·44 per year (6·25–67·50) and cost per DALY averted was $192·30 per year (42·48–506·27; appendix pp 9, 26). Benchmarking against nationally relevant decision rules for Côte d'Ivoire indicates that SET has a 74·0% chance of representing a cost-effective intervention (appendix pp 10–11, 33–34).

## Discussion

In this 2-year trial, children living in the clusters with SET and insecticide-treated nets were 38% less likely to have a malaria case than children living in clusters with insecticide-treated nets alone. There was also a substantial reduction in the number of infective bites, with malaria vector populations and EIRs more than halved indoors and outdoors, in SET clusters compared with control clusters. Numerous laboratory, semi-field, and modelling studies have been suggestive of the potential of SET,^20^, ^21^, ^24^, ^27^ but this is the first trial done at sufficient scale to show an epidemiological effect. These results are encouraging for SET as a novel malaria control intervention.

The lethal house lure approach we tested was a combination of targeted delivery of insecticides using In2Care Eave Tubes with house improvement (screening and general repairs) to reduce mosquito entry. We expected SET to have a better chance of reducing transmission compared with either screening or Eave Tubes alone. We observed a substantial reduction in mosquito densities indoors, but the intervention did not completely prevent mosquito entry, partly because perfect mosquito proofing is difficult to achieve but also because householders do not keep doors and windows closed at all times. However, because of the insecticidal action of the Eave Tubes, mosquitoes could have been killed even after they had entered the house if they attempted to exit through the Eave Tubes.^21^ The contribution of the insecticidal component is further supported by the reduction in mosquito densities that we observed outdoors. An overall reduction in density could benefit the whole community, making SET more equitable, because its effects extend to households that might not be suitable for the intervention themselves.

Coverage of SET was generally good (average of 73% across all clusters), reflecting the good acceptability of the intervention to householders (DTD, unpublished data). Houses did not receive the intervention if the homeowner did not consent, if the occupants did not own the property (ie, rental properties), or if the installation team determined that SET could not be safely installed without damaging the house. This variation resulted in a range of coverages of SET in our clusters, enabling exploratory analyses that suggest a positive relationship between SET coverage and reduction in malaria. This pattern is further supported by data showing that the frequency of clinical malaria was reduced in children living in households without SET if the cluster had more than 70% coverage with SET. These results are consistent with community protection, but the trial was not designed to test this hypothesis.

Although the distribution of insecticide-treated nets in the trial clusters followed National Malaria Control Program guidelines and recipients of SET were requested to continue using their insecticide-treated nets, lower bednet use was still reported in clusters that received SET than in those that did not. Whether this reduced use was due to some perceived benefit of the SET intervention by householders is unknown, but one feature of SET is that it is a passive technology once it is installed, and it protects anyone sleeping in the house without requiring any specific behaviour. Despite universal coverage of insecticide-treated nets in this trial, malaria transmission remained substantial, emphasising the need for new interventions, such as SET, and further combinations of interventions.

We selected a commercially available pyrethroid for this trial. Although central Côte d'Ivoire is an area of intense pyrethroid resistance, early evaluation of candidates found that this product had good efficacy when delivered in an Eave Tube due to a high dose transfer of insecticide powder from the electrostatic netting.^18^, ^22^ Inserts collected from villages reliably produced mortality of more than 80% in bioassays with local mosquitoes up to 4 months following treatment, at which point all inserts were retreated. Persistence of the insecticide under the field trial conditions was shorter than suggested in the initial studies (4 months under village conditions compared with >9 months in earlier laboratory tests and studies of experimental huts).^22^ Although this delivery method was effective against the local mosquito population, and pyrethroids are reliable and safe for malaria vector control with existing regulatory approval across many countries, continued use of pyrethroids is not ideal for insecticide resistance management. Nonetheless, the trial provides essential proof of principle of the novel insecticide delivery system. Much like the early days of insecticide-treated nets, there is potential for further product development to improve performance and explore different active ingredients for use in novel resistance management strategies. This development would need to include a supply chain and business model for the distribution and replacement of inserts, as well as extending the longevity of insecticide on the inserts, work that is being done by In2Care.

Our cost-effectiveness analysis found that, in terms of cost per case averted, SET appears within the range of other key, cost-effective, malaria control interventions. Although the cost per DALY averted exceeds that for other interventions from older studies, it is widely accepted that new interventions are needed, and that these are unlikely to be as effective at driving down transmission as the primary interventions have been. Furthermore, if economies of scale can be achieved in SET (as they are with indoor residual spraying) then the difference between these two interventions will be reduced. Additionally, disentangling the relative contributions of the screening and Eave Tubes, to the overall impact of SET, could provide opportunities for optimisation of the intervention to improve cost-effectiveness. Because the cost of screening is roughly equivalent to the cost of Eave Tubes, if the insecticidal activity alone provides protection from malaria (eg, through mosquito mortality observed in an experimental hut study of Eave Tubes^21^), then there could be an alternative model for deploying Eave Tubes alone.

As with any house improvement intervention, SET is a complex intervention to implement at scale. Despite the effect of housing on health outcomes, housing is not considered the domain of public health, and house improvement does not fit into existing public health product distribution pathways, such as mass drug administration, bednet distributions, or even indoor residual spraying campaigns. Moreover, modification of people's homes can be considered intrusive. Because these challenges are common to any vector control effort targeting the built environment, they are already the focus of ongoing discussions.^6^, ^31^ The emerging consensus is that successful implementation of house-based malaria control interventions will require us to rethink distribution pathways and rely on multisectoral collaborations at every level. Rapid economic and population growth in sub-Saharan Africa means that millions of new houses will be built in the coming decade and millions more existing houses will be retrofitted with modern housing features.^13^ Clear and easy pathways therefore need to be created for the implementation of SET and other house improvement interventions. Failing to move forward on housing and malaria would be a monumental missed opportunity.

## Data sharing

Data collected for the study, including deidentified participant data and data dictionaries, will be shared by the corresponding author upon reasonable request.

## References

[1] Bhatt S, Weiss DJ, Cameron E (2015). The effect of malaria control on *Plasmodium falciparum* in Africa between 2000 and 2015. Nature.

[2] WHO (2019). World malaria report 2019. https://www.who.int/malaria/publications/world-malaria-report-2019/en/.

[3] WHO (2015). Global technical strategy for malaria 2016–2030.

[4] Feachem RGA, Chen I, Akbari O (2019). Malaria eradication within a generation: ambitious, achievable, and necessary. Lancet.

[5] WHO (2019). Malaria eradication: benefits, future scenarios and feasibility. Executive summary, WHO Strategic Advisory Group on Malaria Eradication. https://www.who.int/publications-detail/strategic-advisory-group-malaria-eradication-executive-summary.

[6] von Seidlein L, Wood H, Brittain OS (2019). Knowledge gaps in the construction of rural healthy homes: a research agenda for improved low-cost housing in hot-humid Africa. PLoS Med.

[7] Furnival–Adams J, Olanga EA, Napier M, Garner P (2019). Housing interventions for preventing malaria. Cochrane Database Syst Rev.

[8] Ondiba IM, Oyieke FA, Ong'amo GO, Olumula MM, Nyamongo IK, Estambale BBA (2018). Malaria vector abundance is associated with house structures in Baringo County, Kenya. PLoS One.

[9] Jatta E, Jawara M, Bradley J (2018). How house design affects malaria mosquito density, temperature, and relative humidity: an experimental study in rural Gambia. Lancet Planet Health.

[10] Tusting LS, Ippolito MM, Willey BA (2015). The evidence for improving housing to reduce malaria: a systematic review and meta-analysis. Malar J.

[11] Getawen SK, Ashine T, Massebo F, Woldeyes D, Lindtjørn B (2018). Exploring the impact of house screening intervention on entomological indices and incidence of malaria in Arba Minch town, southwest Ethiopia: a randomized control trial. Acta Trop.

[12] Kirby MJ, Ameh D, Bottomley C (2009). Effect of two different house screening interventions on exposure to malaria vectors and on anaemia in children in The Gambia: a randomised controlled trial. The Lancet.

[13] Tusting LS, Gething PW, Gibson HS (2020). Housing and child health in sub-Saharan Africa: A cross-sectional analysis. PLoS Med.

[14] Killeen GF, Masalu JP, Chinula D (2017). Control of malaria vector mosquitoes by insecticide-treated combinations of window screens and eave baffles. Emerg Infect Dis.

[15] Mmbando AS, Ngowo H, Limwagu A, Kilalangongono M, Kifungo K, Okumu FO (2018). Eave ribbons treated with the spatial repellent, transfluthrin, can effectively protect against indoor-biting and outdoor-biting malaria mosquitoes. Malar J.

[16] Beach RF, Ii TKR, Sexton JD (1993). Effectiveness of permethrin-impregnated bed nets and curtains for malaria control in a holoendemic area of Western Kenya. Am J Trop Med Hyg.

[17] Knols BGJ, Farenhorst M, Andriessen R (2016). Eave tubes for malaria control in Africa: an introduction. Malar J.

[18] Andriessen R, Snetselaar J, Suer RA (2015). Electrostatic coating enhances bioavailability of insecticides and breaks pyrethroid resistance in mosquitoes. Proc Natl Acad Sci.

[19] Sternberg ED, Ng'habi KR, Lyimo IN (2016). Eave tubes for malaria control in Africa: initial development and semi-field evaluations in Tanzania. Malar J.

[20] Snetselaar J, Njiru BN, Gachie B (2017). Eave tubes for malaria control in Africa: prototyping and evaluation against *Anopheles gambiae* s.s. and *Anopheles arabiensis* under semi-field conditions in western Kenya. Malar J.

[21] Barreaux AMG, Brou N, Koffi AA (2018). Semi-field studies to better understand the impact of eave tubes on mosquito mortality and behaviour. Malar J.

[22] Oumbouke WA, Tia IZ, Barreaux AMG (2018). Screening and field performance of powder-formulated insecticides on eave tube inserts against pyrethroid resistant *Anopheles gambiae* s.l.: an investigation into ‘actives’ prior to a randomized controlled trial in Côte d'Ivoire. Malar J.

[23] WHO (2020). Overview of intervention classes and prototype/products under Vector Control Advisory Group (VCAG) review for assessment of public health value. https://apps.who.int/iris/bitstream/handle/10665/274451/WHO-CDS-VCAG-2018.03-eng.pdf?ua=1.

[24] Waite JL, Lynch PA, Thomas MB (2016). Eave tubes for malaria control in Africa: a modelling assessment of potential impact on transmission. Malar J.

[25] Koffi AA, Ahoua Alou LP, Adja MA, Chandre F, Pennetier C (2013). Insecticide resistance status of *Anopheles gambiae* s.s. population from M'Bé: a WHOPES-labelled experimental hut station, 10 years after the political crisis in Côte d'Ivoire. Malar J.

[26] Koffi AA, Ahoua Alou LP, Djenontin A (2015). Efficacy of Olyset® Duo, a permethrin and pyriproxyfen mixture net against wild pyrethroid-resistant *Anopheles gambiae* s.s. from Côte d'Ivoire: an experimental hut trial. Parasite.

[27] Sternberg ED, Cook J, Ahoua Alou LP (2018). Evaluating the impact of screening plus eave tubes on malaria transmission compared to current best practice in central Côte d'Ivoire: a two armed cluster randomized controlled trial. BMC Public Health.

[28] Rushby JF, Hanson K (2001). Calculating and presenting disability adjusted life years (DALYs) in cost-effectiveness analysis. Health Policy Plan.

[29] Pinder M, Conteh L, Jeffries D (2016). The RooPfs study to assess whether improved housing provides additional protection against clinical malaria over current best practice in The Gambia: study protocol for a randomized controlled study and ancillary studies. Trials.

[30] Dalrymple U, Arambepola R, Gething PW, Cameron E. How long do rapid diagnostic tests remain positive after anti-malarial treatment? *Malar J* 2; **17:** 228. 10.1186/s12936-018-2371-9 PMC5994115 29884184

[31] Shenton FC, Addissie A, Alabaster G (2019). Research agenda for preventing mosquito-transmitted diseases through improving the built environment in sub-Saharan Africa. Cities Health.

