# Supplementary material for: Impact and cost-effectiveness of a lethal house lure against malaria transmission in central Côte d'Ivoire: a two-arm, cluster-randomised controlled trial
Source: Lancet. 2021 Feb 27;397(10276):805–15. doi: 10.1016/S0140-6736(21)00250-6 (PMC7910282; doi:10.1016/S0140-6736(21)00250-6)
Supplement: Supplementary appendix [file mmc1.pdf]

THE LANCET

Supplementary appendix

This appendix formed part of the original submission and has been peer reviewed. We post it as supplied by the authors.

Supplement to: Sternberg ED, Cook J, Ahoua Alou LP, et al. Impact and cost-effectiveness of a lethal house lure against malaria transmission in central Côte d'Ivoire: a two-arm, cluster-randomised controlled trial. *Lancet* 2021; **397:** 805–15.

Supplementary appendices to Impact and cost-effectiveness of a lethal house lure against malaria transmission in central Côte d'Ivoire: a two-group, cluster-randomised controlled trial

Appendix 1: Summary of adverse events, authorship as per main paper

Appendix 2: Cost and cost-effectiveness of screening plus Eave Tubes in cluster randomised control trial setting in Côte d'Ivoire. Eve Worrall, Jackie Cook, Eleanore D. Sternberg, Dimi T. Doudou, Matthew B. Thomas

Appendix 1: Summary of adverse events

Children were examined for adverse events at each visit if the child appeared unwell or if the parents/guardians reported the child had been recently unwell. In addition to a physical examination, children (or their guardians) were asked about symptoms in the previous 48 hours. The symptoms that were monitored were: chills, sweats, headache, fatigue, vomiting, cough, nasal discharge, diarrhoea, conjunctivitis, jaundice, indrawing, rattle and catarrh. If children were present for a visit but not examined, they were considered not to have symptoms. Reports of adverse events were generally low. The data for each arm are summarised below. “Number” in the table refers to the total number of child-visits across the monitoring period.

	**ITN arm**		**SET arm**		**Total**	
**Symptom**	**Number**	**%**	**Number**	**%**	**Number**	**%**
**No chills**	35381	97·8	35778	98·4	71159	98·1
**Chills**	788	2·2	573	1·6	1361	1·9
**No sweats**	35974	99·7	36270	99·8	72244	99·7
**Sweats**	117	0·3	71	0·2	188	0·3
**No headache**	33360	93·1	33933	93·8	67293	93·4
**Headache**	2472	6·9	2257	6·2	4729	6·6
**No fatigue**	35365	98·9	35936	99·4	71301	99·1
**Fatigue**	403	1·1	233	0·6	636	0·9
**No vomiting**	35386	97·7	35927	98·7	71313	98·2
**Vomiting**	847	2·3	461	1·3	1308	1·8
**No cough**	35446	97·8	35720	98·2	71166	98.0
**Cough**	809	2·2	669	1·8	1478	2.0
**No discharge**	35687	98·4	35946	98·8	71633	98·6
**Discharge**	570	1·6	443	1·2	1013	1·4
**No diarrhoea**	35950	99·2	36101	99·2	72051	99·2
**Diarrhoea**	303	0·8	288	0·8	591	0·8
**No conjunctivitis**	35835	98·8	36139	99·3	71974	99·1
**Conjunctivitis**	425	1·2	253	0·7	678	0·9
**No jaundice**	36239	99·9	36373	99·9	72612	99·9
**Jaundice**	21	0·1	19	0·1	40	0·1
**No indrawing**	36209	99·9	36343	99·9	72552	99·9
**Indrawing**	50	0·1	49	0·1	99	0·1
**No rattle**	36208	99·9	36338	99·9	72546	99·9
**Rattle**	52	0·1	52	0·1	104	0·1
**No catarrh**	36177	99·8	36325	99·8	72502	99·8
**Catarrh**	83	0·2	66	0·2	149	0·2
**No rash**	36224	99·9	36329	99·8	72553	99·9
**Rash**	36	0·1	63	0·2	99	0·1

Appendix 2: Costs and cost-effectiveness of screening plus Eave Tubes in Côte d'Ivoire

Eve Worrall[1], Jackie Cook[2], Eleanore D. Sternberg[1,3], Dimi T. Doudou[4], Matthew B. Thomas[3,5]

1. Department of Vector Biology, Liverpool School of Tropical Medicine, Liverpool, United Kingdom

2. MRC Tropical Epidemiology Group, Department of Infectious Disease Epidemiology, London School of Hygiene and Tropical Medicine.

3. Department of Entomology, Center for Infectious Disease Dynamics, The Pennsylvania State University, University Park, PA, USA.

4. Université Alassane Ouattara, Bouaké, Côte d'Ivoire.

5. York Environmental Sustainability Institute and Department of Biology, University of York, York, UK

*Correspondence on this appendix should be sent to Eve.Worrall@lstmed.ac.uk

Summary

Malaria vector control has contributed substantially to the significant reductions in malaria over recent years, and insecticide treated nets (ITNs) have been widely adopted and scaled up across sub-Saharan Africa. Malaria control interventions have been shown to be highly cost-effective in a variety of settings, with ITNs being among the most cost-effective interventions in global health. New malaria control interventions are required to meet ambitious malaria elimination targets, reduce the health and economic burden of malaria, and overcome the challenges of drug and insecticide resistance. Given the scarcity of health care resources, competing priorities, and shrinking malaria budgets, new interventions must be highly cost-effective. We therefore conducted a cost-effectiveness analysis to measure the incremental (marginal) economic and financial cost per malaria case and disability adjusted life year (DALY) averted of adding Screening and Eave Tubes (SET) to ITNs, compared to ITNs alone from the societal (provider and community) and provider perspective. The potential cost-effectiveness of SET at scale over time was simulated to facilitate comparison with other malaria control interventions and cost-effectiveness benchmarks.

Objectives

The primary objective of the economics study component was to measure the incremental cost-effectiveness, in terms of cost per case and cost per DALY averted, of adding SET to ITNs from the societal and (separately) provider perspective.

The secondary objectives were to: (i) measure the relative contributions to cost of the distinct programme elements namely: screening installation and initial housing modifications (Screening); Eave Tube installation (Eave Tube), retreatment/replacement of the insecticide on the Eave Tubes (Retreatment) and repeat rounds of housing modification repairs (Housing Modification Repairs), (ii) identify the inputs that contribute most to overall costs (cost drivers) and (iii) estimate the potential cost-effectiveness of providing SET at large scale over an extended period under operational conditions.

Methods

Identifying and defining activities

We conducted activity-based costing using the ingredients approach. First, we identified the activities conducted, next, we identified and categorised the inputs used during these activities, then we valued them in terms of their economic and financial costs. We identified activities and inputs via visits to the trial site during implementation, review of project documents and discussions with project staff. Activities were defined as either implementation or research, and the cost of research activities were excluded from the analysis.

Capturing and analysing costs

We devised a costing spreadsheet to capture relevant data including: programme element, activity, cost type (capital/non-capital), useful life of item, proportion allocated to each element, partner/institution incurring the cost, major cost category (cost category 1), sub-cost category (cost category 2) and detailed cost category (cost category 3), unit type, unit quantity and unit price. All expenditure was recorded in the currency used, most often the West African Franc (XOF) or Euro (€). XOF costs were converted to Euros and then all costs were converted to 2018 US Dollars ($) using mean exchange rates (XOF to Euro and Euro to US Dollar) for the period 01 September 2016 to 30 April 2019 (0·00152449 and 1·1443 respectively)(1).

We obtained financial cost estimates for each input from the financial records of implementing partners. Community members were not paid for participation in the trial and there were no financial costs to them. We identified donated insecticide and community inputs from project records and estimated their economic costs using market rates, with insecticide being included in provider and societal economic costs, and community inputs included in societal economic costs only. The social science team that conducted ethnographic research alongside the trial identified community inputs (e.g. time, labour, materials), and, together with the economist, these were converted to their economic value using appropriate market rates. Intangible community costs, for example infringement on religious beliefs or creation of discord in communities or households, were identified but not quantified in economic terms. We categorised costs as either capital or non-capital according to the above definition. In the financial cost analysis, we

annualised capital costs by dividing them by their estimated useful life. In the economic cost analysis, we annualised capital using the useful life and discount rate of 3%. We calculated total costs by cost type and for each intervention element. Where inputs were shared between programme elements (e.g. equipment used for housing modification and Eave Tube installation), a proportion of the cost was allocated to each, based on proxies and or discussions with relevant colleagues. We calculated total cost per house by dividing the total cost by the number of houses covered with the SET intervention.

Cost-effectiveness analysis

We conducted cost-effectiveness modelling in @Risk software version 7.5 add-in (2) for Excel (3) to estimate the potential cost and cost-effectiveness of providing SET at scale over an extended period of years under operational scenarios. To compare the cost-effectiveness of interventions with different useful life (e.g. annual IRS versus SET or ITNs which may last multiple years) and different multi-year cost profiles (i.e. year one costs not equal to subsequent years' costs), it is necessary to standardise both costs and outcomes. We therefore made assumptions about the useful life of each element of the SET intervention and used this in combination with measured costs to generate estimates of the average annual cost per SET house and per person protected for a programme lasting multiple years.

We combined societal or provider perspective economic costs, useful life estimates, demographic data, and epidemiological outcomes (malaria cases averted) from the trial with health and demographic parameters (malaria case fatality rate, proportion and duration of cases that are severe or otherwise and life expectancy data for Côte d'Ivoire) to estimate cost per case and DALY averted for a base-case, worst and best case, with worse and best judged based on intervention cost-effectiveness. We also calculated cost per malaria death averted for comparison with other studies. We calculated DALYs according to standard methods using WHO life tables, without age weighting or discounting (so-called 'no frills DALY') and separately, with 3% discounting on life years gained (i.e. 3% discount rate implies that a health year of life gained 10 years from now is worth 24% less than a year gained now) with no age weights.

Sensitivity Analysis

We explored uncertainty and model sensitivity using Monte Carlo simulations (100,000 iterations) with input parameters set as either a point estimate (known variables) or range/distribution function (Table 2). We calculated average annual cost per case and DALY averted (and, for completeness/comparison purposes, cost per house and person protected, and cost per death averted) as a point estimate (the mean of 100,000 iterations) with uncertainty represented by the cut-off value where the 5% and 95% of simulation outputs fall (90% credible interval). We conducted sensitivity analysis to rank and quantify inputs (e.g. cost or useful life) according to the amount of swing (positive or negative) they caused on the outputs i.e. cost-effectiveness indicators cost per case and cost per DALY averted. We conducted the sensitivity analysis in @Risk version 7.5 (2), which sorted all iterations of the simulation in ascending order of the relevant input, divided them into ten bins and calculated the mean output value for each bin. Inputs were then ranked by the range between their highest value for each bin and their lowest value and presented as tornado charts showing overall change in output value through the range of input values. We used simple tornado charts to show how the output mean (cost per case or DALY averted) was affected by changes in the input values (e.g. people per house) ranked by the most to least important input. We used the sequential contribution to variance technique to calculate how much more of the variance in each output was explained by adding each of a sequence of inputs to a regression model, and presented these results on a combined tornado chart for both outputs.

Presentation and interpretation of results

In the text we report annualised (at 3%), societal perspective costs unless indicated otherwise, however not-annualised and provider perspective costs are included in tables for completeness. Cost per case, DALY, and death averted, are calculated using annualised economic costs from societal and provider perspective, with and without discounting of DALYs. Cost-effectiveness estimates are shown for the point estimate of the mean incremental cost divided by the mean incremental cases or DALYs averted, with the 90% credible interval shown in brackets. Cost per DALY averted was interpreted against three decision rules: the frequently used (but widely disputed) threshold of one-times and three-times gross domestic product (GDP) per capita (4) (denoted henceforth as GDPx1 and GDPx3 respectively), and the more conservative, empirically derived cost per DALY averted for the Ivorian health system produced by Ochalek et al (5) which represents a country specific estimate of the opportunity costs associated with additional

health care costs (denoted henceforth as Op. Cost). Results of the simulated provider cost per DALY averted are plotted on the cost-effectiveness plane and interpreted against GDPx1, GDPx3 and Op. Cost, and a cost-effectiveness acceptability curve (CEAC) is used to summarise the impact of uncertainty on the results of the evaluation by plotting the probability of cost-effectiveness against a range of possible values of the cost-effectiveness threshold and the decision rules. All results are in 2018 United States Dollars (US$).

Ethics

Ethical approval for the economics work was obtained under the auspices of the main ethical approval for CRT activities in the USA and in Côte d'Ivoire. The trial was reviewed and approved by the Côte d'Ivoire Ministry of Health ethics committee (039/MSLS/CNER-dkn), the Pennsylvania State University's Human Research Protection Program under the Office for Research Protections (STUDY00003899 and STUDY00004815), and the London School of Hygiene and Tropical Medicine ethical review board (No.11223). We obtained informed consent from all trial participants or guardians for participants under the age of 18. A trial steering committee monitored trial progress and adherence to protocol.

Results

Activities and programme elements

The implementation activities included in the analysis by institutions or partner involved responsible are shown in Table 1. Programme elements screening (S) and Eave Tube (ET) installation were implemented simultaneously once between September 2016 and March 2017. At the same time, eaves were closed, and repairs were made to walls, doors and windows in an attempt to mosquito proof houses. During ET installation, untreated inserts were placed in the tubes. Subsequently, inserts were treated and retreated with insecticide and housing modification repairs (HMR) were conducted. Insert treatment (IT) with insecticide took place in a facility rented for the purpose, field teams then took the treated inserts to the houses where the untreated (first time) or used (subsequently) inserts were replaced with treated inserts (Insert Replacement [IR]). Used inserts were returned to the treatment facility where they were cleaned and stored ready for the next retreatment round (Insert Cleaning [IC]). At the same time as houses were visited for

IR, houses were assessed to produce a list of any HMR needed, with these being completed on the subsequent IR/HMR visit. Between March 2017 and April 2019 IT, IR and IC were conducted six times (IT1-IT6, IR1-IR6, IC1-IC6) and HMR was conducted five times (HMR1-HMR5). S and ET costs are presented by element, and retreatment (comprising IT, IR and IC) and HMR are grouped into six rounds (Rounds 1-6) for presentation of results (

**Not-annualised**																
**Cost type**	**Economic**								**Financial**							
**Element Perspective**	**Screening**	**%**	**Eave Tube**	**%**	**Rounds 1-6**	**%**	**Total**	**%**	**Screening**	**%**	**Eave Tube**	**%**	**Rounds 1-6**	**%**	**Total**	**%**
**Provider**	256,105·28	36·7	273,037·80	39·1	169,393·47	24·2	698,536·55	100	256,105·28	37·1	273,037·80	39·5	161,861·52	23·4	691,004·60	100
**Community**	35,979·66	49·5	36,764·68	50·5	-	-	72,744·34	100	-	-	-	-	-	-	-	-
**Societal**	292,084·95	37·9	309,802·48	40·2	169,393·47	22·0	771,280·89	100	256,105·28	37·1	273,037·80	39·5	161,861·52	23·4	691,004·60	100
**Annualised**																
**Provider**	253,586·47	39·0	248,773·25	38·2	148,317·01	22·8	650,676·72	100	253,507·97	39·3	252,005·39	39·0	140,128·21	21·7	645,641·57	100
**Community**	35,979·66	49·5	36,764·68	50·5	-	-	72,744·34	100	-	-	-	-	-	-	-	-
**Societal**	289,566·13	40·0	285,537·93	39·5	148,317·01	20·5	723,421·06	100	253,507·97	39·3	252,005·39	39·0	140,128·21	21·7	645,641·57	100

Table 6 Economic and financial cost by round (rounds 1 to round 6), societal perspective* 2018 US$

Cost type	Economic						Financial					
**Round Number and cost type**	**Round 1**	**Round 2**	**Round 3**	**Round 4**	**Round 5**	**Round 6**	**Round 1**	**Round 2**	**Round 3**	**Round 4**	**Round 5**	**Round 6**
**Not-annualised**	24,755.93	32,964.52	23,463.20	24,872.77	29,675.25	33,661.79	24,755.93	28,837.19	23,463.20	21,468.15	29,675.25	33,661.79
**Annualised**	21,243.19	29,451.78	19,950.46	21,360.03	26,162.51	30,149.04	21,133.72	25,214.98	19,840.99	17,845.93	26,053.03	30,039.57

*Societal and provider perspective equal as no community costs incurred

Table 7 Cost by cost type, category and element (screening, Eave Tube and rounds1-6) annualised economic and financial costs, societal (this page) and provider (next page) perspective (US$2018)

		Economic								Financial							
		Screening		Eave Tube		Round1-6		Total		Screening		Eave Tube		Round1-6		Total	
Cost type and category		$	%	$	%	$	%	$	%	$	%	$	%	$	%	$	%
Societal Perspective																	
Non-capital	Labour	139,865·84	48·3	107,049·69	37·5	55,792·87	37·6	302,708·40	41·8	134,691·08	53·1	101,874·92	40·4	55,792·87	39·8	292,358·87	45·3
	Materials/consumable	47,924·59	16·6	24,645·80	8·6	4,153·43	2·8	76,723·82	10·6	47,575·70	18·8	24,296·90	9·6	4,153·43	3·0	76,026·03	11·8
	Accommodation	29,945·74	10·3	29,945·74	10·5	11,650·71	7·9	71,542·20	9·9	0	0·0	0	0·0	10,727·74	7·7	10,727·74	1·7
	Transport	26,113·50	9·0	16,695·52	5·8	33,803·09	22·8	76,612·11	10·6	26,113·50	10·3	16,695·52	6·6	33,803·09	24·1	76,612·11	11·9
	Freight	23,060·10	8·0	24,395·59	8·5	0	0·0	47,455·69	6·6	23,060·10	9·1	24,395·59	9·7	0	0·0	47,455·69	7·4
	Tools and equipment	11,256·09	3·9	11,560·50	4·0	8,617·06	5·8	31,433·65	4·3	11,256·09	4·4	10,775·49	4·3	8,617·06	6·1	30,648·63	4·7
	Netting	5,384·79	1·9	0	0·0	0	0·0	5,384·79	0·7	5,384·79	2·1	0	0·0	0	0·0	5,384·79	0·8
	Management fee	4,128·06	1·4	6,456·71	2·3	0	0·0	10,584·78	1·5	4,128·06	1·6	6,456·71	2·6	0	0·0	10,584·78	1·6
	Storage	457·92	0·2	457·92	0·2	0	0·0	915·85	0·1	0	0·0	0	0·0	0	0·0	0	0·0
	Communication	52·33	0·0	52·33	0·0	0	0·0	104·67	0·0	0	0·0	0	0·0	0	0·0	0	0·0
	Tubes and inserts	0	0·0	53,146·34	18·6	0	0·0	53,146·34	7·3	0	0·0	53,146·34	21·1	0	0·0	53,146·34	8·2
	Insecticide	0	0·0	0	0·0	19,753·06	13·3	19,753·06	2·7	0	0·0	0	0·0	13,144·09	9·4	13,144·09	2·0
	Other	0	0·0	0	0·0	3,023·30	2·0	3,023·30	0·4	0	0·0	0	0·0	3,023·30	2·2	3,023·30	0·5
	Sub-total	288,188·98	99·5	274,406·15	96·1	136,793·51	92·2	699,388·64	96·7	252,209·32	99·5	237,641·47	94·3	129,261·56	92·2	619,112·35	95·9
Capital	Tools and equipment	1,377·15	0·5	3,461·51	1·2	1,688·12	1·1	6,526·79	0·9	1,298·66	0·5	3,264·21	1·3	1,591·90	1·1	6,154·76	1·0
	Custom tools	0	0·0	7,670·27	2·7	9,835·37	6·6	17,505·63	2·4	0	0·0	11,099·71	4·4	9,274·75	6·6	20,374·46	3·2
	Sub-total	1,377·15	0·5	11,131·78	3·9	11,523·49	7·8	24,032·42	3·3	1,298·66	0·5	14,363·92	5·7	10,866·65	7·8	26,529·22	4·1
Total		289,566·13	100	285,537·93	100	148,317·01	100	723,421·06	100	253,507·97	100	252,005·39	100	140,128·21	100	645,641·57	100

		Economic								Financial							
		Screening		Eave Tube		Round1-6		Total		Screening		Eave Tube		Round1-6		Total	
**Cost type and category**		$	%	$	%	$	%	$	%	$	%	$	%	$	%	$	%
**Provider perspective**																	
Non-capital	**Labour**	134,691·08	53·1	101,874·92	41·0	55,792·87	37·6	292,358·87	44·9	134,691·08	53·1	101,874·92	40·4	55,792·87	39·8	292,358·87	45·3
	**Materials/consumable**	47,575·70	18·8	24,296·90	9·8	4,153·43	2·8	76,026·03	11·7	47,575·70	18·8	24,296·90	9·6	4,153·43	3·0	76,026·03	11·8
	**Accommodation**	0	0·0	0	0·0	11,650·71	7·9	11,650·71	1·8	0	0·0	0	0·0	10,727·74	7·7	10,727·74	1·7
	**Transport**	26,113·50	10·3	16,695·52	6·7	33,803·09	22·8	76,612·11	11·8	26,113·50	10·3	16,695·52	6·6	33,803·09	24·1	76,612·11	11·9
	**Freight**	23,060·10	9·1	24,395·59	9·8	0	0·0	47,455·69	7·3	23,060·10	9·1	24,395·59	9·7	0	0·0	47,455·69	7·4
	**Tools and equipment**	11,256·09	4·4	10,775·49	4·3	8,617·06	5·8	30,648·63	4·7	11,256·09	4·4	10,775·49	4·3	8,617·06	6·1	30,648·63	4·7
	**Netting**	5,384·79	2·1	0	0·0	0	0·0	5,384·79	0·8	5,384·79	2·1	0	0·0	0	0·0	5,384·79	0·8
	**Management fee**	4,128·06	1·6	6,456·71	2·6	0	0·0	10,584·78	1·6	4,128·06	1·6	6,456·71	2·6	0	0·0	10,584·78	1·6
	**Storage**	0	0·0	0	0·0	0	0·0	0	0·0	0	0·0	0	0·0	0	0·0	0	0·0
	**Communication**	0	0·0	0	0·0	0	0·0	0	0·0	0	0·0	0	0·0	0	0·0	0	0·0
	**Tubes and inserts**	0	0·0	53,146·34	21·4	0	0·0	53,146·34	8·2	0	0·0	53,146·34	21·1	0	0·0	53,146·34	8·2
	**Insecticide**	0	0·0	0	0·0	19,753·06	13·3	19,753·06	3·0	0	0·0	0	0·0	13,144·09	9·4	13,144·09	2·0
	**Other**	0	0·0	0	0·0	3,023·30	2·0	3,023·30	0·5	0	0·0	0	0·0	3,023·30	2·2	3,023·30	0·5
	**Sub-total**	252,209·32	99·5	237,641·47	95·5	136,793·51	92·2	626,644·30	96·3	252,209·32	99·5	237,641·47	94·3	129,261·56	92·2	619,112·35	95·9
Capital	**Tools and equipment**	1,377·15	0·5	3,461·51	1·4	1,688·12	1·1	6,526·79	1·0	1,298·66	0·5	3,264·21	1·3	1,591·90	1·1	6,154·76	1·0
	**Custom tools**	0	0·0	7,670·27	3·1	9,835·37	6·6	17,505·63	2·7	0	0·0	11,099·71	4·4	9,274·75	6·6	20,374·46	3·2
	**Sub-total**	1,377·15	0·5	11,131·78	4·5	11,523·49	7·8	24,032·42	3·7	1,298·66	0·5	14,363·92	5·7	10,866·65	7·8	26,529·22	4·1
**Total**		253,586·47	100	248,773·25	100	148,317·01	100	650,676·72	100	253,507·97	100	252,005·39	100	140,128·21	100	645,641·57	100

Table 8 Total economic and financial cost per house by element under trial conditions, societal and provider perspective, 2018 US$

Perspective	Cost±	Screening	Eave Tube	Round 1 to 6	Total
Societal	Economic	95·85 (96·68)	94·52 (102·55)	49·10 (56·07)	239·46 (255·31)
	Financial	83·92 (84·78)	83·42 (90·38)	46·38 (53·58)	213·72 (228·73)
Provider	Economic	83·94 (84·78)	82·35 (90·38)	49·10 (56·07)	215·38 (231·23)
	Financial	83·92 (84·78)	83·42 (90·38)	46·38 (53·58)	213·72 (228·73)

±Costs are annualised, not-annualised costs are shown in ( ). Note these costs are as measured under trial conditions and therefore do not have a credibility interval.

Table 9 Simulated cost per house and person protected per year and cost-effectiveness per case, death and DALY averted (with and without discounting of life years gained) 2018US$

Perspective	Cost per unit	Mean cost (and 90% credible interval)
Societal	House per year	56·54 (39·97 to 81·24)
	Person protected per year	21·47 (6·08 to 49·99)
	Case averted	28·91 (6·82 to 74·21)
	Death averted	9,612·05 (2,179·81 to 2,5018·56)
	DALY averted	Life years gained:<br>undiscounted 210·29 (46·16 to 553·57)<br>discounted 392·30 (88·01 to 1,021·16)
Provider	House per year	51·76 (36·77 to 73·43)
	Person protected per year	19·62 (5·59 to 45·38)
	Case averted	26·44 (6·25 to 67·50)
	Death averted	8,792·91 (1,997·43 to 2,2760·68)
	DALY averted	Life years gained:<br>undiscounted 192·30 (42·48 to 506·27)<br>discounted 359.00 (81·01 to 932·01)

Figure 1).

Incremental costs

The total incremental societal cost of delivering all elements of SET was $723,421·06 with the majority ($699,388·64/$723,421·06, 96·7%) attributed to non-capital items and incurred by providers ($650,676·72/$723,421·06, 90.0%) (Table 3). Of the economic resources contributed by communities ($72,744·34), the majority (82·3%) was for board and lodgings for the teams responsible for installing the Eave Tubes and screening, and conducting housing repairs at the start of the trial (Figure 2 and Table 4).

Cost by element

S, ET and the six rounds comprised 40·0%, 39·5%, 20·5% of societal and 39·0%, 38·2% and 22·8% of provider costs respectively. From the community perspective, costs were incurred for S and ET only (49·5% and 50·5% of community costs respectively) (Table 5). Round 1-6 costs ranged between $19,950·46 (R3) and $30,149·04 (R6) with a median cost of $23,761·27 per round (Table 5).

Cost drivers overall and by element

Labour was the main cost driver accounting for 41·8% of societal economic costs, followed by materials and consumables (10·6%), transport (10·6%), accommodation (9·9%) and tubes and inserts (7·3%). Together these cost categories account for 80·3% of economic costs. Insecticide accounts for 2·7% of total societal costs. The top five cost drivers from the provider perspective are labour (44·9%), transport (11·8%), materials and consumables (11·7%), tubes and inserts (8·2%) and freight (7·3%), with insecticide accounting for 3.0% of provider costs (Table 7).

Labour was the major cost driver for each individual element. By element, the second largest cost drivers were: S: materials and consumables (16·6% and 18·8% societal and provider perspective, respectively); ET: tubes and inserts (18·6% and 21·4% societal and provider respectively) and Rounds 1-6: transport (22·8% societal and provider perspective) (Table 7).

Cost per house

The total number of houses receiving the SET intervention was 3021, thus the total economic cost per house was $239·46 and $215·38 from the societal and provider perspectives respectively (Table 8 and Figure 3).

Cost and cost-effectiveness

Societal cost per house and person protected per year were $56·54 (90% credible interval $39·97 to $81·24) and $21·47 ($6·08 to $49·99) respectively, with equivalent provider costs being $51·76 ($36·77 to $73·43) and $19·62 ($5·59 to $45·38) respectively. Societal cost per case and death averted were $28·91 ($6·82 to $74·21) and $9,612·05 ($2,179·81 to $25,018·56) respectively, with equivalent provider costs being $26·44 ($6·25 to $67·50) and $8,792·91 ($1,997·43 to $22,760·68) respectively. Societal cost per DALY averted was $210·29 ($46·16 to $553·57) without discounting of life years gained and $392·30 ($88·01 to $1,021·16) with discounting. Provider cost per DALY averted was $192·30 ($42·48 to $506·27) and $359.00 ($81·01 to $932·01) with and without discounting respectively (Table 9).

Sensitivity analysis

Cost-effectiveness ratios were most sensitive to the estimated number of people per house, with this variable explaining 43·7% and 38·7% of variance in societal cost per case and DALY averted (respectively). Cases averted per person per year and useful life of screening were next most important variables, explaining 17·0% and 15·1% and 4·8% and 4·3% of variance in cost per case and DALY averted respectively. Assumptions around the malaria case fatality rate and years of life lost per death contributed 3·5% and 3·2% of variance in cost per DALY averted (Figure 4 and Figure 5).

Interpretation

Plotting the cost-effectiveness simulation results on the cost-effectiveness plane and decision rules (Figure 6 Top) and examining uncertainty with a CEAC against the Op. Cost decision rules (Figure 6 Bottom) indicates that SET is 100% likely to be cost-effective against GDPx1 and GDPx3 decision rules, and for any threshold greater than or equal to US$2338 per DALY averted. The probability of SET being cost-effective against the Op. Cost decision rule is 74·0% (68·8-79·8% lower and upper estimates).

Discussion

In this trial, the total annualised societal economic cost of delivering all elements of the intervention was $723,421·06, which was mostly attributed to non-capital items and borne by providers rather than communities (90% versus 10%). The annualised and not-annualised costs are very similar due to the low proportion of capital costs. Labour was the main cost driver overall (41·8% and 44·9% societal and provider costs respectively) and within each programme element. This is broadly in line with the range for IRS programmes, where labour costs

range between 22·6-57·1% of economic costs (author calculations using data from (6)). Insecticide costs were a minor percentage of SET costs (2·7% and 3·0% societal and provider respectively). In contrast, insecticide contributes between 18·5-74·7% of IRS programme economic costs (author calculations using data from (6)).

S, and ET comprised roughly equal proportion of costs (40·0%, 39·5%, societal and 39·0%, 38·2% provider, respectively). Under the current trial, the intervention tested was a combination of these two elements, with ITNs, and therefore it is not possible to establish to what extent each element contributed to the health outcomes achieved. A better understanding of the relative contribution of S and ET to the overall impact of SET would provide opportunities for optimising the intervention to improve cost-effectiveness. Though care would be needed to ensure any cost reduction, was not countered by reduced community acceptability leading to lowered effectiveness.

To help inform decision making at country level, we compared SET to three different decision rules, finding that SET has a 100% chance of being cost-effective against the GDPx1 and GDPx3 thresholds and a high likelihood (74·0% probability) of SET being cost-effective when compared with an empirically derived estimate of the plausible cost per DALY averted from changes in health expenditure in Côte d'Ivoire. In other words, SET has a 74.0% chance of representing a cost-effective intervention, compared with existing healthcare activities in Côte d'Ivoire.

Comparison between alternative malaria control tools used throughout sub-Saharan Africa is useful to inform global policy and support resource allocation for further intervention trials; hence, we compare our findings to the existing literature on the cost-effectiveness of malaria control interventions. SET provider cost per case averted was $26·44 (90% credible interval $6·25 to $67·50). According to a systematic review of cost and cost-effectiveness of malaria control interventions (6), ITN cost per malaria case averted ranges between $11-$47 and between $5-146 for IRS[1]. Thus, SET is in the same range as these two widely used interventions. SET provider cost per DALY averted was and $192·30 (90% credible interval $42·48 to $506·27). In the same review (6), ITN cost per DALY averted ranges between $9-$129 and $158-$176 for IRS[2], thus we find that in terms of cost per DALY averted, SET is less cost-effective than ITNs and IRS, but with potential to achieve similar levels of cost-effectiveness as both interventions, if we consider the credible interval on our simulations.

---

[1] Upper and lower range read from chart showing cost per case averted in figure 7 and converted to 2018 US$ for comparison purposes by authors.
[2] As before, data presented by White et al has been adjusted to 2018 US$ for comparison purposes

It should be noted that this is not a perfect comparison, as the studies in the systematic review are out-dated and do not reflect the more recent lower ITN prices and higher insecticide (IRS) prices, different programmatic scale and implementation approaches used for both interventions over recent years. Furthermore, while health outcomes such as DALYs averted are arguably more useful for decision makers than intermediate outcomes such as cases averted, they are subject to additional assumptions that complicate comparisons between studies. This is highlighted in our own sensitivity analysis, where cost per DALY averted is affected by assumptions regarding malaria case fatality rate and years of life lost due to malaria deaths. In addition, different: baseline levels of malaria epidemiology; coverage of, and combinations with, other interventions, will alter effectiveness, further hampering comparison between studies.

In present day malaria endemic Africa, ITNs are the mainstay of malaria vector control. Having a high baseline level of ITN coverage means that the protective efficacy of the next additional intervention is subject to diminishing marginal returns and is unlikely to be as cost-effective as the primary intervention. A comparison between ITN plus SET and ITN plus IRS (the next most commonly used publicly funded vector control tool) is perhaps the most sensible in the current policy context. IRS is both effective and cost-effective, but it is not without practical limitations, including the huge logistical effort required to deliver it on (at least) an annual basis, and the large volumes of insecticides used, which contribute to a substantial proportion of costs and pose health and environmental challenges for safe disposal. The much smaller quantity of insecticides used for SET could potentially be an advantage over IRS. In terms of logistical effort, the initial installation of SET was a complex undertaking, but the subsequent rounds to maintain the intervention were less intensive. This opens the possibility of models that allow retreatment to operate on a commercial basis, once the upfront costs and effort of installing the Eave Tubes has been made.

Like all cost-effectiveness analyses, particularly those based on a single trial, this analysis is subject to several assumptions. While every attempt was made to be conservative and transparent in our analysis, the sensitivity analysis revealed the importance of key variables. In particular, the number of people per house, cases averted and useful life of screening, affected cost per case and DALY averted. We measured (rather than assumed) people per house and cases averted and are therefore confident that the sensitivity of our results reflects a true range rather than uncertainty. The importance of people per house in driving SET cost-effectiveness is analogous with the importance of similar intervention to humans ratios for other interventions (ITNs: people per net; IRS: people per house and larval source management: people protected per area of breeding site treated) which also strongly

influence cost-effectiveness, likely leading to large variations in cost-effectiveness within and between interventions. This highlights the importance of considering how different contexts may affect cost-effectiveness and the need for additional data on the cost and effectiveness of SET in different settings. Given the importance of the assumption of the useful life of screening and (to a lesser extent) ET, additional follow up in the Ivorian trial site would be useful to better understand the useful life of these element in operational conditions. The extent to which modifications to the intervention package (i.e. impact of S and ET) and scale economies could improve cost-effectiveness also requires further investigation.

Trial based cost-effectiveness evaluations, such as the one presented here, have several advantages including readily available cost data, and well-defined carefully measured effectiveness measures. However, a potential limitation is the transferability of the findings from research to a real-life context. We have attempted to address some of these factors using simulations and sensitivity analysis, however in reality the translation from research study to scaled implementation can affect cost-effectiveness in myriad ways. Differences in the availability of resources, intervention quality, intervention coverage and community engagement may all change with scaled-up implementation. This is common with all interventions and was notably seen with ITNs where early trials and evaluations assumed a useful life of five or sometimes even seven years for the nets which, in hindsight, likely over-estimated cost-effectiveness. On the other hand, what was not factored in to early ITN evaluations was the dramatic fall in bednet prices which has, to some degree, countered the optimistic useful life estimates. For SET, like any new technology, the development and potential scale-up will need to be guided by successive evaluations and modifications based on user feedback, scientific studies, and economic evaluation.

The difference between the financial and economic costs in this trial was driven primarily by donated community resources and to a lesser extent, by a small volume of insecticides donated by a commercial supplier. The largest value resource donated by the community was the provision of meals and accommodation to some of the installation team members. This was necessary when it wasn't practical to drive to and from the installation village in a day, and there were no local commercial guest houses. This contributed to intervention feasibility and reduced provider perspective (transport) costs. Another significant community cost is time spent preparing houses prior to SET installation (removing food and covering items) and then sweeping, tidying and replacing goods afterwards. An important cost, borne by women in the communities, is fetching water to mix the cement. Although this forms a small part of the cost share, it is notable that the social science team did not mention this in their analysis (it came out in subsequent discussions) as it is considered normal for women to fetch water in domestic life.

Discussions revealed that during house building, women would sometimes be paid to fetch water and it was these costs that were used to estimate the value of this activity. No community members were paid for contributions made to the intervention, and while male community members contributed time/labour to the intervention, based on the economic analysis and gender norms in the community, it seems likely that most community costs incurred in this trial were borne by women. This is probably also the case for IRS, where homes must be cleared and emptied, children kept out of the way and water is needed for mixing insecticides. However, this labour is rarely included in economic analysis and, to our knowledge the gender perspective on community costs of vector control interventions has not previously been considered.

In spite of the limitations and challenges of comparing different interventions over time, scale and between settings, this trial suggests that SET is likely to be a cost-effective intervention compared with existing Ivorian health interventions and has the potential to be as cost-effective as IRS when added to ITNs, with a number of possible advantages, and it should be explored further in a range of settings.

References

1. European Central Bank. Euro foreign exchange reference rates [Internet]. Euro foreign exchange reference rates. [cited 2020 Apr 30]. Available from: reference: European Central Bank ECB, Euro foreign exchange reference rates· Available from https://www·ecb·europa·eu/stats/policy_and_exchange_rates/euro_reference_exchange_rates/html/eurofxref-graph-usd·en·html#

2. Palisades Corporation. @Risk Software Version 7.5 [Internet]. New York, USA; Available from: www.palisade.com

3. Microsoft Excel. Microsoft Corporation;

4. Marseille E, Larson B, Kazi DS, Kahn JG, Rosen S. Thresholds for the cost – effectiveness of interventions : alternative approaches. Bull World Health Organ. 2015;93(October 2014):118–24.

5. Ochalek J, Lomas J, Claxton K. Estimating health opportunity costs in low-income and middle-income countries: A novel approach and evidence from cross-country data. BMJ Glob Heal. 2018;3(6).

6. White MT, Conteh L, Cibulskis R, Ghani AC. Costs and cost-effectiveness of malaria control interventions--a systematic review. Malar J [Internet]. 2011/11/05. 2011;10:337. Available from: http://www.ncbi.nlm.nih.gov/pubmed/22050911

7. Oumbouke WA, Tia IZ, Barreaux AMG, Koffi AA, Sternberg ED, Thomas MB, et al. Screening and field performance of powder-formulated insecticides on eave tube inserts against pyrethroid resistant Anopheles gambiae s.l.: An investigation into “actives” prior to a randomized controlled trial in Côte d’Ivoire 11 Medical and Health Scien. Malar J [Internet]. 2018;17(1):1–9. Available from: https://doi.org/10.1186/s12936-018-2517-9

8. WHO. WHO | The World malaria report 2018 [Internet]. Who. 2018. 22 p. Available from: www.who.int/malaria%0Ahttps://apps.who.int/iris/bitstream/handle/10665/275867/9789241565653-eng.pdf?ua=1%0Ahttps://www.who.int/malaria/publications/world-malaria-report-2018/en/; consulté le 22/03/2019%0Ahttps://www.who.int/malaria/media/world-malaria-rep

9. Salomon JA, Haagsma JA, Davis A, de Noordhout CM, Polinder S, Havelaar AH, et al. Disability weights for the Global Burden of Disease 2013 study. Lancet Glob Heal. 2015;3(11):e712–23.

10. Global Health Observatory data repository. Life tables by country Côte d'Ivoire [Internet]. Life tables by year. 2016. Available from: https://apps.who.int/gho/data/view.main.LT62040?lang=en

Table 1 Main implementation and research activities of institutions and partners involved with the study

Institution/Partner	Implementation Activities	Research Activities<br>(Excluded from analysis)
Community in CRT area	Attend and participate in community sensitisation activities<br>Provide space for workshops during installation<br>Prepare homes for installation<br>Provide access to their homes during installation	Collaboration with data collection (surveys, focus group discussions) and allowing interventions to be installed in their homes
Penn State University (PSU)		Coordinate and manage cluster randomised trial (CRT)<br>Ensure ethical standards<br>Report to funder<br>Conduct social science research
In2Care, Wageningen, The Netherlands, (commercial company) a partner in the development of Eave Tube technology	Manufacture and supply Eave Tube inserts<br>Procure and ship equipment and supplies (e.g. tools, nails, wall plugs) for housing improvement and installation<br>Providing technical and managerial oversight of installation and retreatment<br>Develop bespoke equipment to improve the processes of Eave Tube installation and re/treatment<br>Coordinate community sensitization activities<br>Subcontract Farmstrong (see below)	
Farmstrong, Ivorian/Swiss NGO, subcontracted by In2Care	Make housing improvements (screening and other repairs)<br>Install Eave Tubes<br>Treat inserts with insecticide<br>Install and replace insecticide treated inserts<br>Repairs window screens and housing<br>Recruit, manage, pay trades people and supervisors<br>Supply vehicles to transport field teams<br>Financial and project management	
Institut Pierre Richet (IPR) / Institut National de anté Publique (INSP), Bouaké, Côte d'Ivoire	Facilities used to store equipment and as a base for meetings/trainings·	Clinical oversight of CRT entomological and epidemiological monitoring
Université Alassane Ouattara, Bouaké, Côte d'Ivoire		Design and conduct social science research
London School of Hygiene and Tropical Medicine		Design and conduct epidemiological research
Liverpool School of Tropical Medicine		Design and conduct economics research

Table 2 Input variables used in simulation modelling

Input category	Variable name	Value				Justification		
		**Base case**	**Worst case◊**	**Best case◊**	**Distribution function□**	**Base case**	**Worst case***	**Best Case**
Annualised economic cost	Screening	Societal 289,566.13<br>Provider 253,586.47	Societal 289,566.13<br>Provider 253,586.47	Societal 202,696.29<br>Provider 177,510.53	Triangle (best, base, worst)	Measured cost (trial)	Trial costs assumed upper limit	Assume 30% cost reduction from base case
	Eave Tube	Societal 285,537.93<br>Provider 248,773.25	Societal 285,537.93<br>Provider 248,773.25	Societal 199,876.55<br>Provider 174,141.27	Triangle (best, base, worst)	Measured cost (trial)	Trial costs assumed upper limit	Assume 30% cost reduction from base case
	Housing modification repair per round	Societal and Provider 5,576.51	Societal and provider 8,632.49	Societal and provider 1,759.58	Triangle (best, base, worst)	Measured cost (trial)	Maximum observed cost per round (trial)	Minimum observed cost per round (trial)
	Retreatment per round	Societal and provider 18,676.74	Societal and provider 22,110.03	Societal and provider 18,109.19	Triangle (best, base, worst)	Measured cost (trial)	Maximum observed cost per round (trial)	Minimum observed cost per round (trial)
Useful life (years)	Screening	3	1	10	Triangle (worst, base, best)	Assumption	Assumption	Assumption
	Eave Tube	6	2	10	Triangle (worst, base, best)	Assumption	Assumption	Assumption
	Housing modification repairs (HMR)	0·4	0·08	1·00	Triangle (worst, base, best)	5 HMR rounds conducted post installation over 24 month period (trial)	Assumption: 12 rounds per year	Assumption: single repair round per year
	Retreatment	0·33	0·33	0·75	Triangle (worst, base, best)	6 retreatment rounds conducted post installation over 24 month period (trial)	Assumption: 6 rounds per year as in trial	Useful life of 9 months with dry formulation (7)
Demographic data	People per house	6·14	1·00	10·00	Log Normal, mean 6·14, (max truncated at 10)	Distributed fitted to trial census data, houses with >10 residents recoded as 10 for conservative estimate	Assumption: 1 person per house	Assumption: 10 person per house
Intervention coverage	Houses covered	3,021	3,021	3,021	Point estimate	Observed	n.a.	n.a.
Intervention efficacy	Malaria cases averted per person per year	0·86	0·40	1·24	Uniform	Trial data: control arm mean malaria case incidence per/person/year minus intervention arm mean case incidence per/person/year Table 2 in main paper)	Trial data, lower bound on 95% confidence interval reduction in malaria/cases/person /year control versus intervention	Trial data, upper bound on 95% confidence interval reduction in malaria/cases/perso n/year control versus intervention
DALY input	Malaria case fatality rate (all ages) %	0·283950	0·202740	0·438008	Triangle (worst, base, best)	Calculated by authors using point estimates, Côte d'Ivoire malaria deaths/malaria cases. Source: (8)	Calculated by authors using point estimates, Côte d'Ivoire malaria deaths/malaria cases. Source: (8)	Calculated by authors using point estimates, Côte d'Ivoire malaria deaths/malaria cases. Source: (8)

	Proportion of cases that become severe	0·060	0·050	0·070	Uniform	Assumption	Assumption	Assumption
	Duration of uncomplicated malaria morbidity (days)	3	1	5	Uniform	Assumption	Assumption	Assumption
	Duration of severe malaria morbidity (days)	7	4	12	Uniform	Assumption	Assumption	Assumption
	DALY weight uncomplicated malaria (infectious disease moderate)	0·051	0·032	0·074	Triangle (worst, base, best)	Point estimate DALY weight for Infectious disease, acute episode. Source: (9)	Min of 95% uncertainty range, DALY weight for Infectious disease, acute episode. Source: (9)	Max of 95% uncertainty range on DALY weight for Infectious disease, acute episode. Source: (9)
	DALY weight severe malaria (infectious disease moderate)	0·133	0·088	0·190	Triangle (worst, base, best)	Point estimate DALY weight for Infectious disease, acute episode. Source: (9)	Min of 95% uncertainty range, DALY weight for Infectious disease, acute episode. Source: (9)	Max of 95% uncertainty range, DALY weight for Infectious disease, acute episode.
	Years of life lost per death Discounted at 3% shown in ( )	54.52 (26·84)	29.03 (19·38)	55.71 (27·07)	Triangle (worst, base, best)	(Discounted) M/mean of expected additional years of life for age groups <1 to 10-14 years old, Côte d'Ivoire. Source: (10)	(Discounted) M/mean of expected additional years of life for age groups <1 to 85+ years old, Côte d'Ivoire. Source: (10)	(Discounted) M/mean of expected additional years of life for age groups <1 to 5-9 years old, Côte d'Ivoire. Source: (10)

☼Held constant as scale economies captured in costs not intervention coverage

◊Defined in terms of impact on cost-effectiveness of intervention

□While normal or beta distributions are commonly used to represent uncertainty, we opted to use more simplistic distribution functions (triangle or uniform) to clearly distinguish where assumptions (rather than data) were used to parameterise the distribution function in the sensitivity analysis.

Table 3 Total costs of the intervention by cost type (capital and non-capital) and perspective (Provider, Community, Societal) 2018 US$

	Cost (not-annualised)				Annualised Cost			
**Perspective and cost type**	**Economic**	**% Total**	**Financial**	**% Total**	**Economic**	**% Total**	**Financial**	**% Total**
**Provider**								
Non-capital	626,644·30	89·7%	619,112·35	89·6%	626,644·30	96·3%	619,112·35	95·9%
Capital	71,892·25	10·3%	71,892·25	10·4%	24,032·42	3·7%	26,529·22	4·1%
Total	698,536·55	100·0%	691,004·60	100·0%	650,676·72	100·0%	645,641·57	100·0%
**Community**								
Non-capital	72,744·34	100·0%	0	0	72,744·34	100·0%	0	0
Capital	0	0	0	0	0	0	0	0
Total	72,744·34	100·0%	0	0	72,744·34	100·0%	0	0
**Societal**								
Non-capital	699,388·64	90·7%	619,112·35	89·6%	699,388·64	96·7%	619,112·35	95·9%
Capital	71,892·25	9·3%	71,892·25	10·4%	24,032·42	3·3%	26,529·22	4·1%
**Total**	771,280·89	100·0%	691,004·60	100·0%	723,421·06	100·0%	645,641·57	100·0%

Table 4 Economic value of resources donated to the programme by the community (2018 US$)

Cost category 1	Cost category 2	Description of donated resources	Resource valuation method	Economic cost ($)+	%
**Board and lodging**	Installation Team accommodation	Housing of work teams by chiefs in host families including meals	Estimated number of person nights' accommodation provided by households multiplied by cost of local accommodation e.g. rural guest houses	54,118·81	74·4
	Installation Team meals	Households/villages provide food to workers	This happened daily in almost all villages with communities providing and cooking (on fires needing fuel that had to be fetched or purchased) meals for the field teams· Estimated based on number of person days of casual labour spent in the field multiplied by cost of a local meal	5,772·67	7·9
**Labour**	Time before, during and after installation	Almost all households took practical steps in preparation for installation such as covering or move food and furniture, move personal effects to avoid sand and sawdust, then cleaning and returning items after installation· Keeping children safe and away from installation teams/installation sites during installation· In some cases, household members also provided labour to installation process	Approximate number of households (3000) multiplied by 1-day labour at national minimum wage rate for Côte d'Ivoire	9,578·99	13·2
	Time for libation ceremony	Household/community practice to obtain permission for intervention from household spirits/deities	Assume done in all households but with large variation in time spent (15mins – 2 hours). Take midpoint of time estimate and value using local agricultural wage rate, equivalent to National Minimum wage in Côte d'Ivoire	614·39	0·8
	Women's labour (fetching water)	Women's labour to fetch water for the cement mixing· Water carrying is exclusively done by women in these communities	The quantity of water was obtained by calculating amount of water needed to mix the cement reported in the financial costs· The cost per litre of water fetched (e.g. for building work was used).	156·15	0·2
**Storage**	Of equipment and materials	Households used to store equipment overnight by field teams	This was a substantial input from communities and some complained due to the time items were stored· Imputed costs multiplied by duration of field activity by village and a mid-point cost from range provided by social science team	915·85	1·3
**Materials and Consumables**	Sand	Households asked to provide sand to the bricklayers (2000 francs per wheelbarrow of sand) donated	Happened infrequently so assumed 1 per village at indicated cost	697·79	1·0
**Tools and equipment**	Small equipment	Loan of additional equipment when tools being used by other installation team (e.g. hammer, wheelbarrow)	Quantity of some frequently borrowed equipment increased to cover community loan	785·01	1·1
**Communication**	Town criers	Village chiefs mobilised criers (bards or keepers of oral history) to inform population about installation teams	Number of criers used and for how long multiplied by wage rate paid to criers by census research team	104·67	0·1
**Intangible**	Change in home environment	Changes in temperature, light levels and air flow within households which can be positive or negative· Perception of smell and side effects even prior to treatment with insecticide	Intangible cannot be valued in monetary terms	Not quantified	-
	Challenging beliefs or disturbing harmony	Infringement on religious beliefs, creation of discord between householders and/or community members who disagree on installation of Eave Tubes	Intangible cannot be valued in monetary terms	Not quantified	-
			Total	72,744·34	100

+annualised and not-annualised costs are the same as no capital costs incurred by community

Table 5 Economic and financial cost by element (Screening, Eave Tube, Rounds 1-6) and perspective (provider, community and perspective) 2018 US$

**Not-annualised**																
**Cost type**	**Economic**								**Financial**							
**Element Perspective**	**Screening**	**%**	**Eave Tube**	**%**	**Rounds 1-6**	**%**	**Total**	**%**	**Screening**	**%**	**Eave Tube**	**%**	**Rounds 1-6**	**%**	**Total**	**%**
**Provider**	256,105·28	36·7	273,037·80	39·1	169,393·47	24·2	698,536·55	100	256,105·28	37·1	273,037·80	39·5	161,861·52	23·4	691,004·60	100
**Community**	35,979·66	49·5	36,764·68	50·5	-	-	72,744·34	100	-	-	-	-	-	-	-	-
**Societal**	292,084·95	37·9	309,802·48	40·2	169,393·47	22·0	771,280·89	100	256,105·28	37·1	273,037·80	39·5	161,861·52	23·4	691,004·60	100
**Annualised**																
**Provider**	253,586·47	39·0	248,773·25	38·2	148,317·01	22·8	650,676·72	100	253,507·97	39·3	252,005·39	39·0	140,128·21	21·7	645,641·57	100
**Community**	35,979·66	49·5	36,764·68	50·5	-	-	72,744·34	100	-	-	-	-	-	-	-	-
**Societal**	289,566·13	40·0	285,537·93	39·5	148,317·01	20·5	723,421·06	100	253,507·97	39·3	252,005·39	39·0	140,128·21	21·7	645,641·57	100

Table 6 Economic and financial cost by round (rounds 1 to round 6), societal perspective* 2018 US$

Cost type	Economic						Financial					
**Round Number and cost type**	**Round 1**	**Round 2**	**Round 3**	**Round 4**	**Round 5**	**Round 6**	**Round 1**	**Round 2**	**Round 3**	**Round 4**	**Round 5**	**Round 6**
**Not-annualised**	24,755.93	32,964.52	23,463.20	24,872.77	29,675.25	33,661.79	24,755.93	28,837.19	23,463.20	21,468.15	29,675.25	33,661.79
**Annualised**	21,243.19	29,451.78	19,950.46	21,360.03	26,162.51	30,149.04	21,133.72	25,214.98	19,840.99	17,845.93	26,053.03	30,039.57

*Societal and provider perspective equal as no community costs incurred

Table 7 Cost by cost type, category and element (screening, Eave Tube and rounds1-6) annualised economic and financial costs, societal (this page) and provider (next page) perspective (US$2018)

		Economic								Financial							
		Screening		Eave Tube		Round1-6		Total		Screening		Eave Tube		Round1-6		Total	
Cost type and category		$	%	$	%	$	%	$	%	$	%	$	%	$	%	$	%
Societal Perspective																	
Non-capital	Labour	139,865·84	48·3	107,049·69	37·5	55,792·87	37·6	302,708·40	41·8	134,691·08	53·1	101,874·92	40·4	55,792·87	39·8	292,358·87	45·3
	Materials/consumable	47,924·59	16·6	24,645·80	8·6	4,153·43	2·8	76,723·82	10·6	47,575·70	18·8	24,296·90	9·6	4,153·43	3·0	76,026·03	11·8
	Accommodation	29,945·74	10·3	29,945·74	10·5	11,650·71	7·9	71,542·20	9·9	0	0·0	0	0·0	10,727·74	7·7	10,727·74	1·7
	Transport	26,113·50	9·0	16,695·52	5·8	33,803·09	22·8	76,612·11	10·6	26,113·50	10·3	16,695·52	6·6	33,803·09	24·1	76,612·11	11·9
	Freight	23,060·10	8·0	24,395·59	8·5	0	0·0	47,455·69	6·6	23,060·10	9·1	24,395·59	9·7	0	0·0	47,455·69	7·4
	Tools and equipment	11,256·09	3·9	11,560·50	4·0	8,617·06	5·8	31,433·65	4·3	11,256·09	4·4	10,775·49	4·3	8,617·06	6·1	30,648·63	4·7
	Netting	5,384·79	1·9	0	0·0	0	0·0	5,384·79	0·7	5,384·79	2·1	0	0·0	0	0·0	5,384·79	0·8
	Management fee	4,128·06	1·4	6,456·71	2·3	0	0·0	10,584·78	1·5	4,128·06	1·6	6,456·71	2·6	0	0·0	10,584·78	1·6
	Storage	457·92	0·2	457·92	0·2	0	0·0	915·85	0·1	0	0·0	0	0·0	0	0·0	0	0·0
	Communication	52·33	0·0	52·33	0·0	0	0·0	104·67	0·0	0	0·0	0	0·0	0	0·0	0	0·0
	Tubes and inserts	0	0·0	53,146·34	18·6	0	0·0	53,146·34	7·3	0	0·0	53,146·34	21·1	0	0·0	53,146·34	8·2
	Insecticide	0	0·0	0	0·0	19,753·06	13·3	19,753·06	2·7	0	0·0	0	0·0	13,144·09	9·4	13,144·09	2·0
	Other	0	0·0	0	0·0	3,023·30	2·0	3,023·30	0·4	0	0·0	0	0·0	3,023·30	2·2	3,023·30	0·5
	Sub-total	288,188·98	99·5	274,406·15	96·1	136,793·51	92·2	699,388·64	96·7	252,209·32	99·5	237,641·47	94·3	129,261·56	92·2	619,112·35	95·9
Capital	Tools and equipment	1,377·15	0·5	3,461·51	1·2	1,688·12	1·1	6,526·79	0·9	1,298·66	0·5	3,264·21	1·3	1,591·90	1·1	6,154·76	1·0
	Custom tools	0	0·0	7,670·27	2·7	9,835·37	6·6	17,505·63	2·4	0	0·0	11,099·71	4·4	9,274·75	6·6	20,374·46	3·2
	Sub-total	1,377·15	0·5	11,131·78	3·9	11,523·49	7·8	24,032·42	3·3	1,298·66	0·5	14,363·92	5·7	10,866·65	7·8	26,529·22	4·1
Total		289,566·13	100	285,537·93	100	148,317·01	100	723,421·06	100	253,507·97	100	252,005·39	100	140,128·21	100	645,641·57	100

		Economic								Financial							
		Screening		Eave Tube		Round1-6		Total		Screening		Eave Tube		Round1-6		Total	
**Cost type and category**		**$**	**%**	**$**	**%**	**$**	**%**	**$**	**%**	**$**	**%**	**$**	**%**	**$**	**%**	**$**	**%**
**Provider perspective**																	
**Non-capital**	**Labour**	134,691·08	53·1	101,874·92	41·0	55,792·87	37·6	292,358·87	44·9	134,691·08	53·1	101,874·92	40·4	55,792·87	39·8	292,358·87	45·3
	**Materials/consumable**	47,575·70	18·8	24,296·90	9·8	4,153·43	2·8	76,026·03	11·7	47,575·70	18·8	24,296·90	9·6	4,153·43	3·0	76,026·03	11·8
	**Accommodation**	0	0·0	0	0·0	11,650·71	7·9	11,650·71	1·8	0	0·0	0	0·0	10,727·74	7·7	10,727·74	1·7
	**Transport**	26,113·50	10·3	16,695·52	6·7	33,803·09	22·8	76,612·11	11·8	26,113·50	10·3	16,695·52	6·6	33,803·09	24·1	76,612·11	11·9
	**Freight**	23,060·10	9·1	24,395·59	9·8	0	0·0	47,455·69	7·3	23,060·10	9·1	24,395·59	9·7	0	0·0	47,455·69	7·4
	**Tools and equipment**	11,256·09	4·4	10,775·49	4·3	8,617·06	5·8	30,648·63	4·7	11,256·09	4·4	10,775·49	4·3	8,617·06	6·1	30,648·63	4·7
	**Netting**	5,384·79	2·1	0	0·0	0	0·0	5,384·79	0·8	5,384·79	2·1	0	0·0	0	0·0	5,384·79	0·8
	**Management fee**	4,128·06	1·6	6,456·71	2·6	0	0·0	10,584·78	1·6	4,128·06	1·6	6,456·71	2·6	0	0·0	10,584·78	1·6
	**Storage**	0	0·0	0	0·0	0	0·0	0	0·0	0	0·0	0	0·0	0	0·0	0	0·0
	**Communication**	0	0·0	0	0·0	0	0·0	0	0·0	0	0·0	0	0·0	0	0·0	0	0·0
	**Tubes and inserts**	0	0·0	53,146·34	21·4	0	0·0	53,146·34	8·2	0	0·0	53,146·34	21·1	0	0·0	53,146·34	8·2
	**Insecticide**	0	0·0	0	0·0	19,753·06	13·3	19,753·06	3·0	0	0·0	0	0·0	13,144·09	9·4	13,144·09	2·0
	**Other**	0	0·0	0	0·0	3,023·30	2·0	3,023·30	0·5	0	0·0	0	0·0	3,023·30	2·2	3,023·30	0·5
	**Sub-total**	252,209·32	99·5	237,641·47	95·5	136,793·51	92·2	626,644·30	96·3	252,209·32	99·5	237,641·47	94·3	129,261·56	92·2	619,112·35	95·9
**Capital**	**Tools and equipment**	1,377·15	0·5	3,461·51	1·4	1,688·12	1·1	6,526·79	1·0	1,298·66	0·5	3,264·21	1·3	1,591·90	1·1	6,154·76	1·0
	**Custom tools**	0	0·0	7,670·27	3·1	9,835·37	6·6	17,505·63	2·7	0	0·0	11,099·71	4·4	9,274·75	6·6	20,374·46	3·2
	**Sub-total**	1,377·15	0·5	11,131·78	4·5	11,523·49	7·8	24,032·42	3·7	1,298·66	0·5	14,363·92	5·7	10,866·65	7·8	26,529·22	4·1
**Total**		253,586·47	100	248,773·25	100	148,317·01	100	650,676·72	100	253,507·97	100	252,005·39	100	140,128·21	100	645,641·57	100

Table 8 Total economic and financial cost per house by element under trial conditions, societal and provider perspective, 2018 US$

Perspective	Cost±	Screening	Eave Tube	Round 1 to 6	Total
Societal	Economic	95·85 (96·68)	94·52 (102·55)	49·10 (56·07)	239·46 (255·31)
	Financial	83·92 (84·78)	83·42 (90·38)	46·38 (53·58)	213·72 (228·73)
Provider	Economic	83·94 (84·78)	82·35 (90·38)	49·10 (56·07)	215·38 (231·23)
	Financial	83·92 (84·78)	83·42 (90·38)	46·38 (53·58)	213·72 (228·73)

±Costs are annualised, not-annualised costs are shown in ( ). Note these costs are as measured under trial conditions and therefore do not have a credibility interval.

Table 9 Simulated cost per house and person protected per year and cost-effectiveness per case, death and DALY averted (with and without discounting of life years gained) 2018US$

Perspective	Cost per unit	Mean cost (and 90% credible interval)
Societal	House per year	56·54 (39·97 to 81·24)
	Person protected per year	21·47 (6·08 to 49·99)
	Case averted	28·91 (6·82 to 74·21)
	Death averted	9,612·05 (2,179·81 to 2,5018·56)
	DALY averted	Life years gained:<br>undiscounted 210·29 (46·16 to 553·57)<br>discounted 392·30 (88·01 to 1,021·16)
Provider	House per year	51·76 (36·77 to 73·43)
	Person protected per year	19·62 (5·59 to 45·38)
	Case averted	26·44 (6·25 to 67·50)
	Death averted	8,792·91 (1,997·43 to 2,2760·68)
	DALY averted	Life years gained:<br>undiscounted 192·30 (42·48 to 506·27)<br>discounted 359.00 (81·01 to 932·01)

Figure 1 Timing and grouping of intervention elements by round number as used in economic analysis

<table>
<tr><th>Sep-16</th><th>Oct-16</th><th>Nov-16</th><th>Dec-16</th><th>Jan-17</th><th>Feb-17</th><th>Mar-17</th><th>Apr-17</th><th>May-17</th><th>Jun-17</th><th>Jul-17</th><th>Aug-17</th><th>Sep-17</th><th>Oct-17</th><th>Nov-17</th><th>Dec-17</th><th>Jan-18</th><th>Feb-18</th><th>Mar-18</th><th>Apr-18</th><th>May-18</th><th>Jun-18</th><th>Jul-18</th><th>Aug-18</th><th>Sep-18</th><th>Oct-18</th><th>Nov-18</th><th>Dec-18</th><th>Jan-19</th><th>Feb-19</th><th>Mar-19</th><th>Apr-19</th></tr>
<tr><td colspan="7">SET [174]</td><td></td><td></td><td></td><td></td><td></td><td></td><td></td><td></td><td></td><td></td><td></td><td></td><td></td><td></td><td></td><td></td><td></td><td></td><td></td><td></td><td></td><td></td><td></td><td></td><td></td></tr>
<tr><td></td><td></td><td></td><td></td><td></td><td></td><td colspan="3">IT1[61]</td><td></td><td colspan="2">IT2[41]</td><td></td><td></td><td></td><td colspan="2">IT3[41]</td><td></td><td></td><td colspan="2">IT4[39]</td><td></td><td></td><td></td><td></td><td colspan="2">IT5[38]</td><td></td><td></td><td></td><td colspan="2">IT6[54]</td></tr>
<tr><td></td><td></td><td></td><td></td><td></td><td></td><td colspan="3">IR1[39]</td><td></td><td colspan="2">IR2[40]</td><td></td><td></td><td></td><td colspan="2">IR3[31]</td><td></td><td></td><td></td><td colspan="4">IR4[90]</td><td></td><td colspan="2">IR5[37]</td><td></td><td></td><td></td><td colspan="2">IR6[40]</td></tr>
<tr><td></td><td></td><td></td><td></td><td></td><td></td><td></td><td></td><td>IC1[20]</td><td></td><td colspan="2">IC2[45]</td><td colspan="4">IC3[99]</td><td colspan="4">IC4[75]</td><td></td><td></td><td colspan="3">IC5[68]</td><td></td><td></td><td colspan="3">IC6[81]</td><td></td><td></td></tr>
<tr><td></td><td></td><td></td><td></td><td></td><td></td><td></td><td></td><td></td><td colspan="2">HMR1
[25]</td><td></td><td></td><td></td><td></td><td colspan="2">HMR2
[31]</td><td></td><td>HMR3[12]</td><td></td><td></td><td></td><td></td><td></td><td></td><td colspan="2">HMR4
[37]</td><td></td><td></td><td></td><td colspan="2">HMR5
[40]</td></tr>
<tr><td></td><td></td><td></td><td></td><td></td><td></td><td colspan="3">Round1</td><td colspan="4">Round2</td><td></td><td></td><td colspan="2">Round3</td><td></td><td colspan="7">Round4</td><td colspan="2">Round5</td><td colspan="5">Round6</td></tr>
</table>

SET Installation of screening and Eave Tube; IT Insert treatment; IR Insert replacement; IC Insert Cleaning; HMR Housing modification repairs; R Round; Number in [ ] indicates number of days each activity took

Figure 2 Breakdown of community resources contributed to intervention

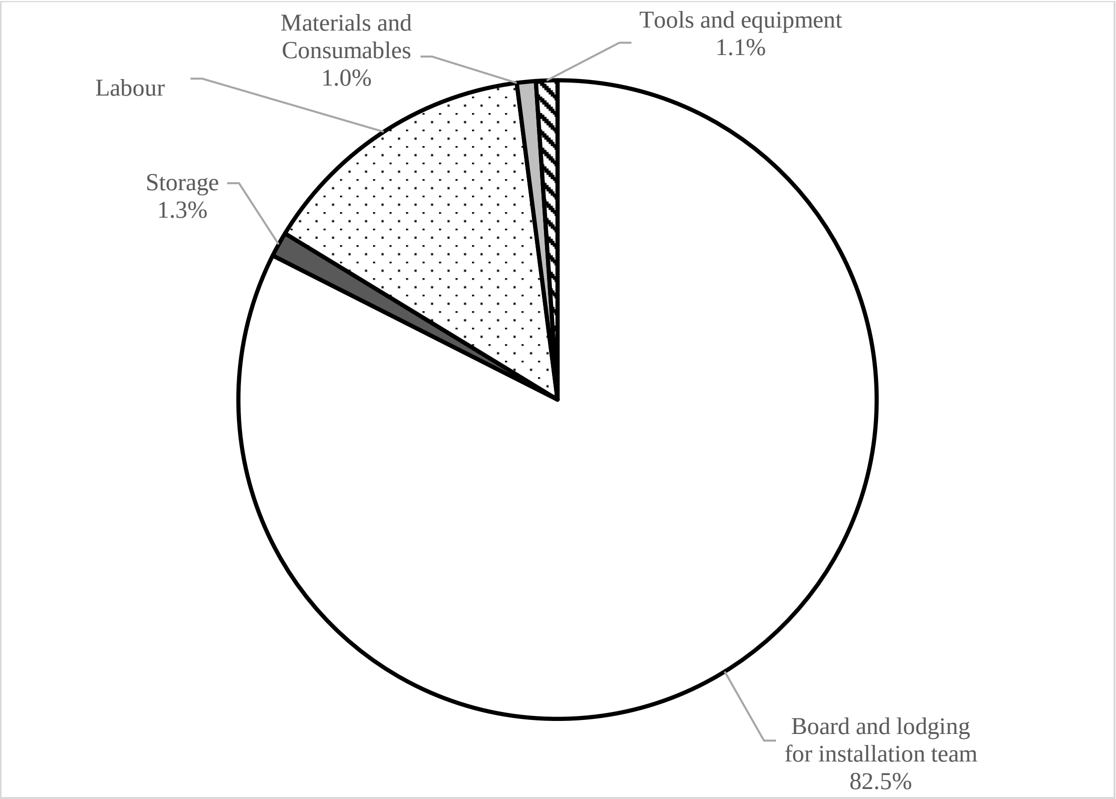

Figure 3 Cost per house by intervention element and perspective US$2018

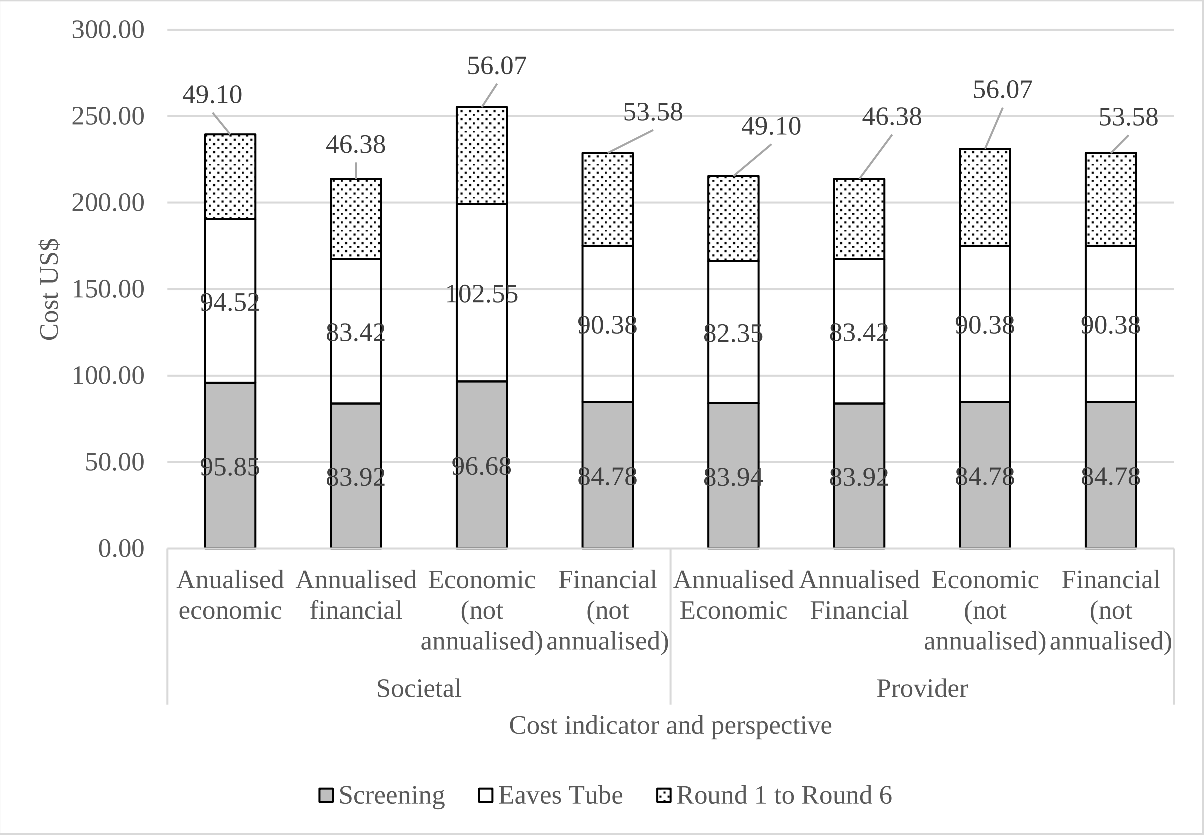

Figure 4 Output (top: Societal cost per case averted, Bottom: Societal cost per DALY averted) sensitivity analysis

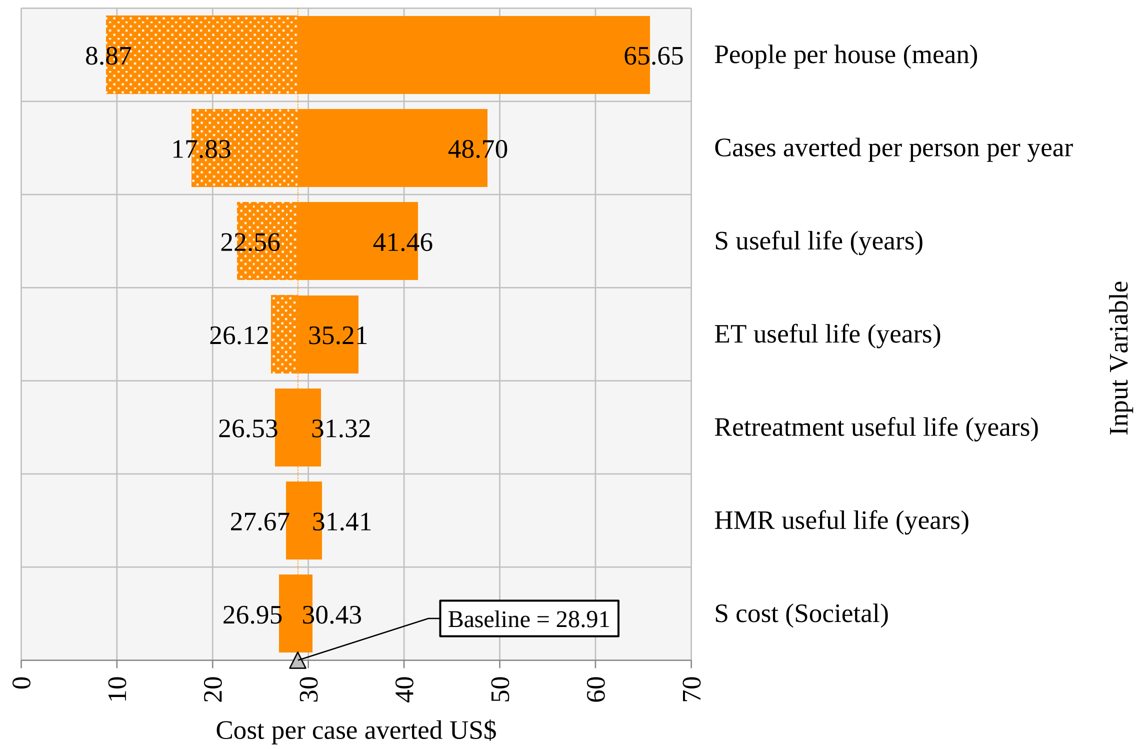

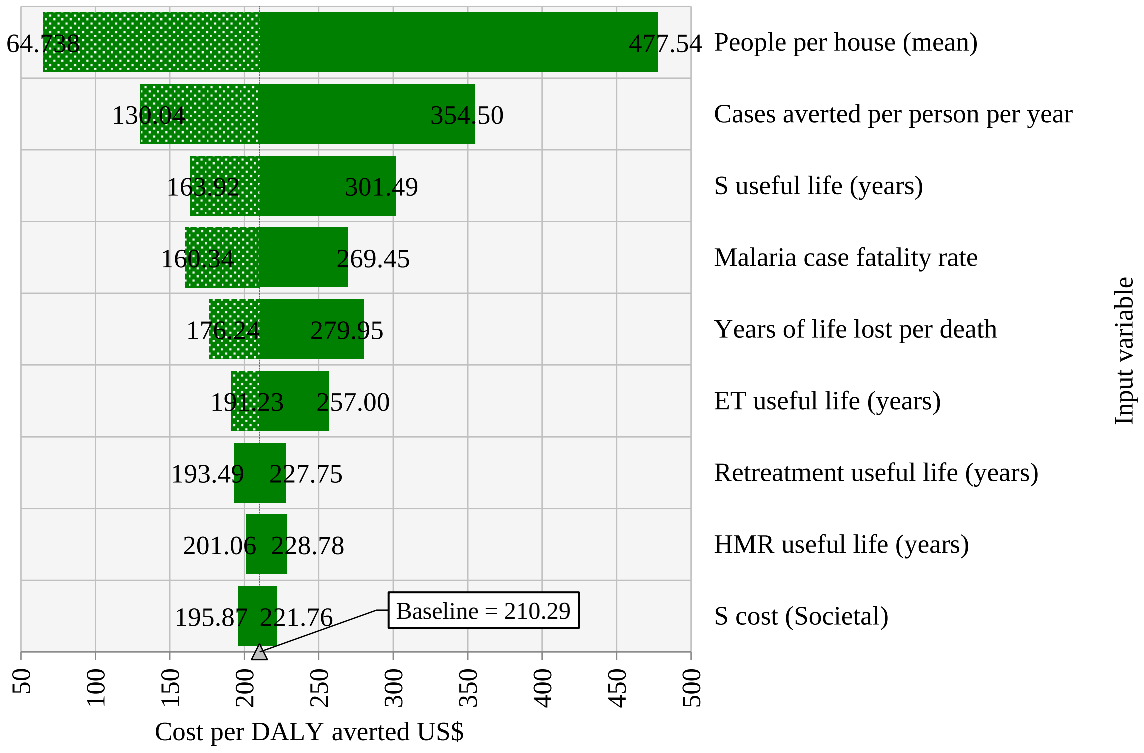

Interpretation: horizontal axis shows cost per case averted with the triangle indicating baseline value of output. Vertical axis shows input variable. For each input variable the bar shows the lower (left hand side) and upper (right hand side) value of the output generated with the simulated range for each input variable. Shading is used to indicate a high (dot shaded) and low (solid fill) value for each input variable. Inputs ranked by their impact on output (top to bottom), only input values with a >10% impact on outputs shown.
DALY Disability adjusted life year; ET Eave Tube; HMR housing modification repairs; S Screening

Figure 5 Sensitivity Analysis

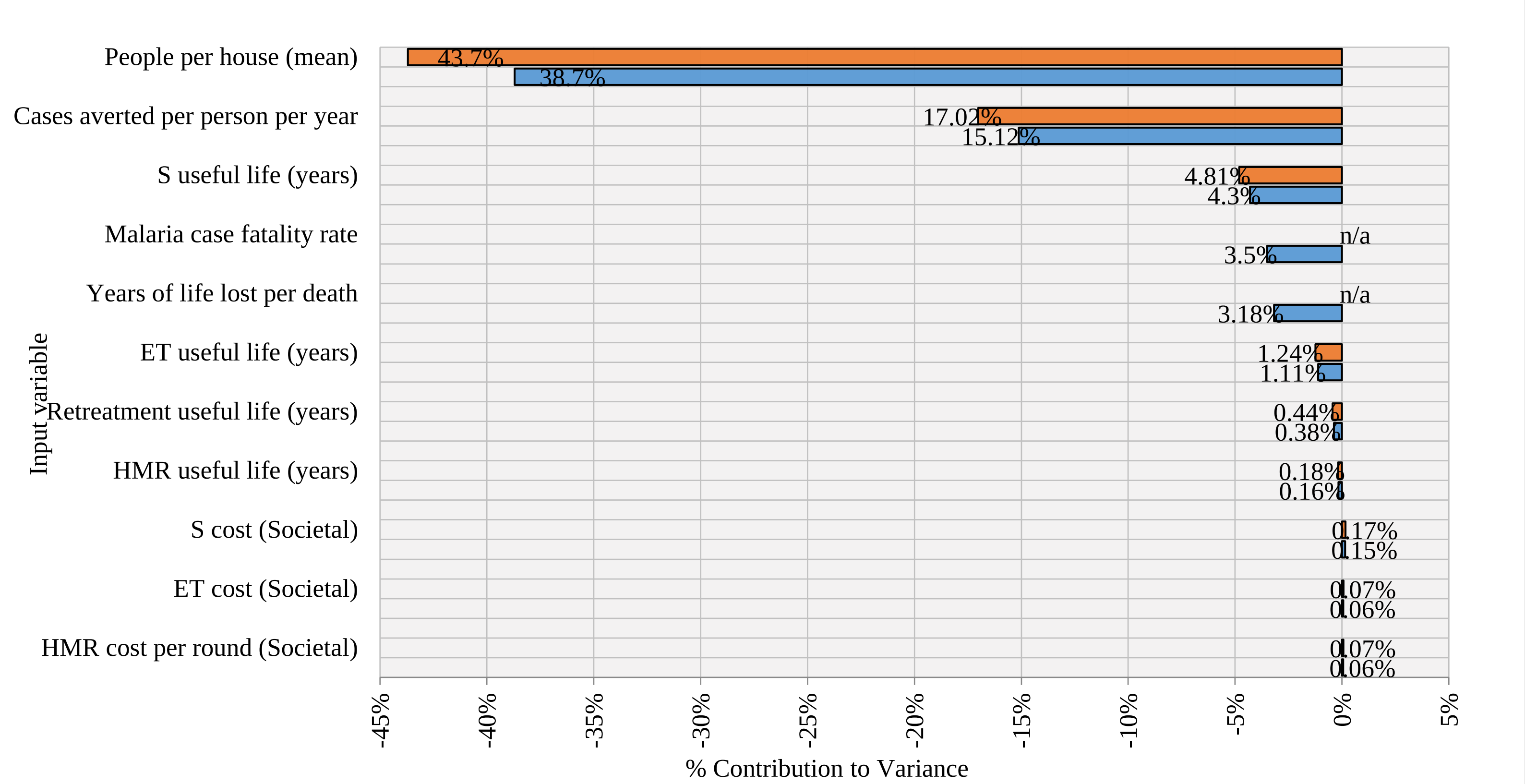

Interpretation: Horizontal access shows % contribution to variance in outputs (orange/upper bar: societal cost per case averted, blue/lower bar: societal cost per disability adjusted life year (DALY) averted caused by each input (vertical axis). Inputs are ranked, top (highest) to bottom (lowest) based on their contribution to variance in societal cost per case averted. The length of each bar illustrates the amount of change in the output attributable to each input. All inputs are shown regardless of magnitude of contribution to variance. ET Eave Tube; DALY Disability adjusted life year; HMR Housing modification repairs; S Screening; n/a not applicable meaning that this input variable is not included in the modelled output variable.

Figure 6 Simulated SET cost per DALY averted against thresholds and estimated opportunity cost shown on the cost-effectiveness plane (top) and as a cost-effectiveness acceptability curve (bottom)

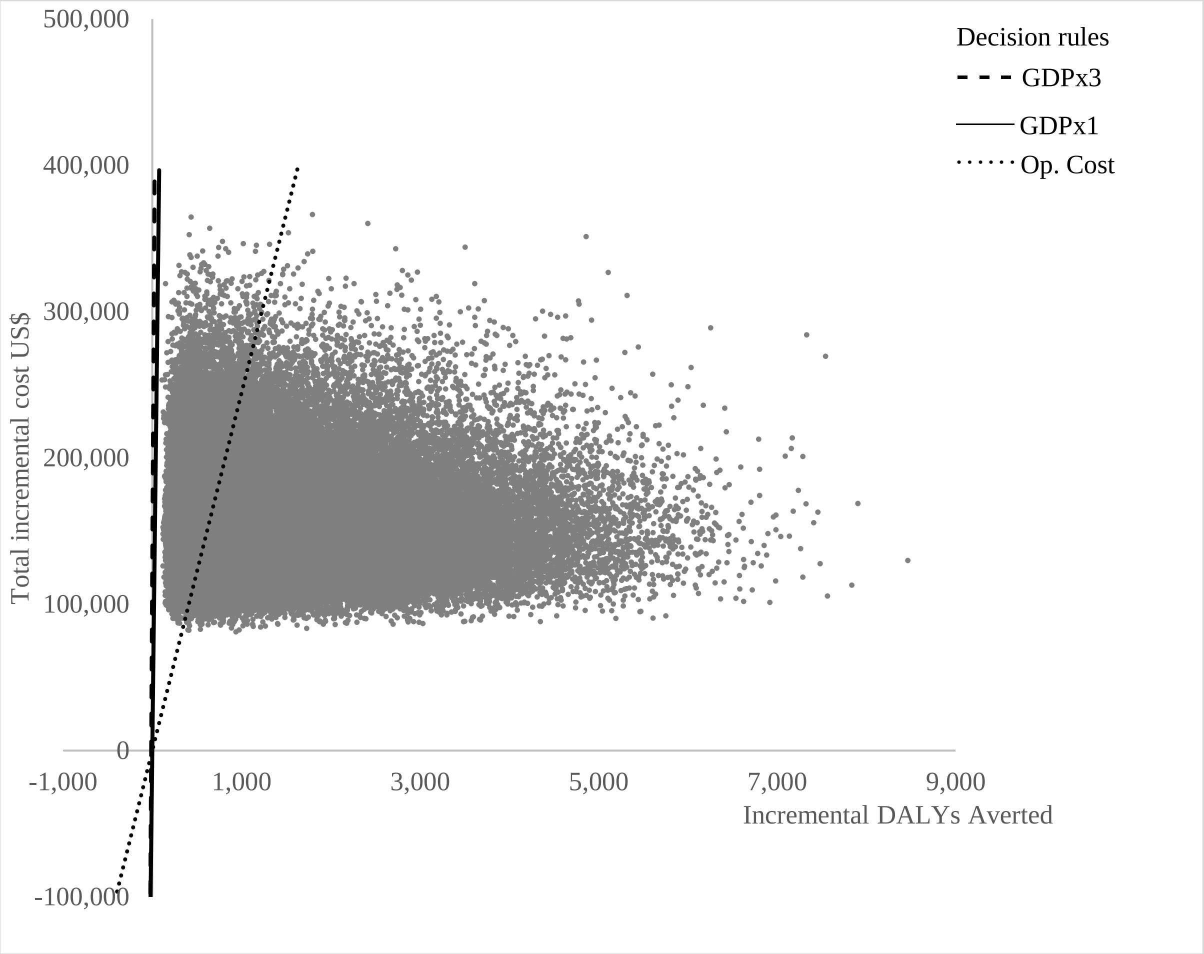

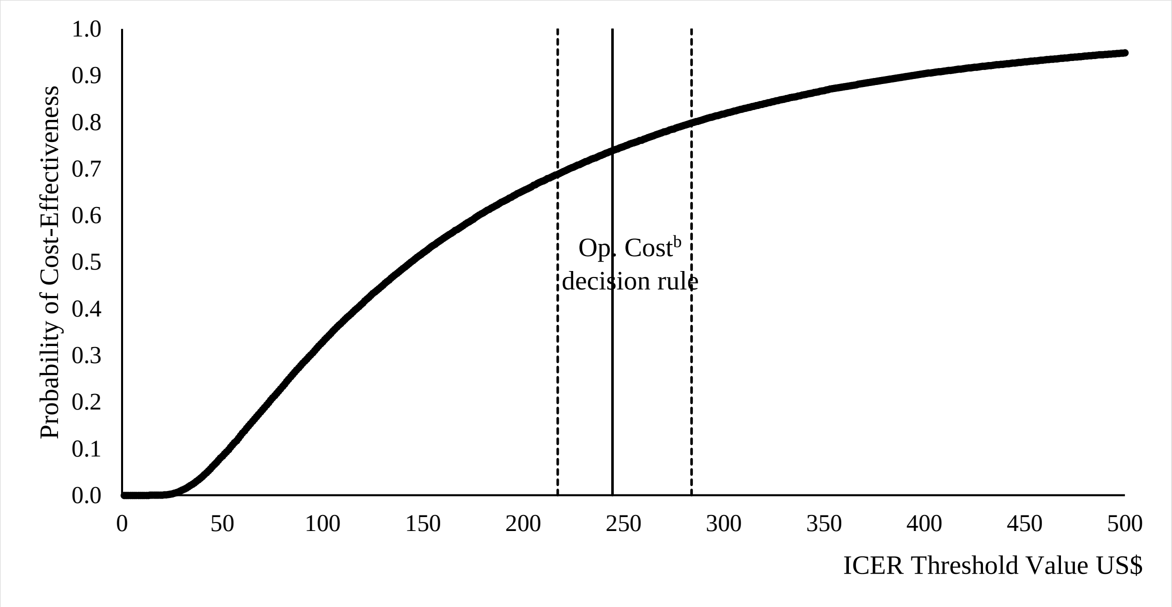

Interpretation: Top panel: Cost-effectiveness plane shows scatter plot of simulated total cost of intervention against DALYs averted for the range of input variables in Table 2. The upper-right quadrant of the cost-effectiveness plane represents outcomes that are both more costly and more effective than current practice. To aid interpretation, various decision rules are applied, illustrated by the three lines. The dashed line represents a

threshold of three-times per capita GDP[a] (GPDx3), the solid line represents a threshold of one-times per capita GDP (GDPx1) and the dotted line represents the opportunity cost of additional health care expenditure (Op. Cost) from (5). Scatter points to the right of the lines are acceptable according to these decision rules. All simulation outputs are acceptable according to GDPx1 and GDPx3 thresholds and the majority are acceptable according to the Op. Cost rule which is explored further in the bottom panel.

Bottom panel: Cost-effectiveness acceptability curve (CEAC) shows the probability that the incremental cost-effectiveness ratio (ICER) i.e. the cost-per DALY averted, is cost-effective against a range of values. In this chart the mean, lower and upper Op. Cost estimates are applied shown by the solid (mean) and dotted (upper and lower) vertical lines. SET has a 74% (68.8-79.8%) chance of being cost-effective against this decision rule. SET has a 100% chance of being cost-effective at any decision threshold of ≤ US$2338 per DALY averted (not shown as it is off the scale of the chart).

[a]GDP Gross Domestic Product per capita for Cote d'Ivoire in 2018 ($5028.92, expressed in 2017 US$) was obtained from World Bank, World Development Indicators (https://data.worldbank.org/indicator/ [accessed 01/07/2020)) and adjusted to 2018 Dollars by authors.

[b]Op. Cost (Opportunity Cost) is the empirically derived cost per DALY averted for the Ivorian health system produced by Ochalek et al (5) which represents an country specific estimate of the opportunity costs associated with additional health care costs. We adjusted the four different cost per DALY averted estimates presented by Ochalek from 2017 US$, to 2018 US$ and used them to calculate the mean, lower and upper estimates plotted on the CEAC chart, $244.47 ($217.19-$283.93) respectively.
